# The HmrABCX pathway regulates the transition between motile and sessile lifestyles in *Caulobacter crescentus* by a mechanism independent of *hfiA* transcription

**DOI:** 10.1128/mbio.01002-24

**Published:** 2024-09-04

**Authors:** Sébastien Zappa, Cécile Berne, Robert I. Morton III, Gregory B. Whitfield, Jonathan De Stercke, Yves V. Brun

**Affiliations:** 1 Département de microbiologie, infectiologie et immunologie, Université de Montréal, Montréal, Quebec, Canada; 2 Department of Biology, Indiana University, Bloomington, Indiana, USA; University of Michigan-Ann Arbor, Ann Arbor, Michigan, USA

**Keywords:** biofilms, flagellar motility, phosphorelay, *Caulobacter crescentus*, signal transduction, cyclic GMP, adhesins

## Abstract

During its cell cycle, the bacterium *Caulobacter crescentus* switches from a motile, free-living state, to a sessile surface-attached cell. During this coordinated process, cells undergo irreversible morphological changes, such as shedding of their polar flagellum and synthesis of an adhesive holdfast at the same pole. In this work, we used genetic screens to identify genes involved in the regulation of the transition from the motile to the sessile lifestyle. We identified a predicted hybrid histidine kinase that inhibits biofilm formation and promotes the motile lifestyle: HmrA (holdfast and motility regulator A). Genetic screens and genomic localization led to the identification of additional genes that form a putative phosphorelay pathway with HmrA. We postulate that the Hmr pathway acts as a rheostat to control the proportion of cells harboring a flagellum or a holdfast in the population. Further genetic analysis suggests that the Hmr pathway impacts c-di-GMP synthesis through the diguanylate cyclase DgcB pathway. Our results also indicate that the Hmr pathway is involved in the regulation of motile to sessile lifestyle transition as a function of various environmental factors: biofilm formation is repressed when excess copper is present and derepressed under non-optimal temperatures. Finally, we provide evidence that the Hmr pathway regulates motility and adhesion without modulating the transcription of the holdfast synthesis regulator HfiA.

**IMPORTANCE:**

Complex communities attached to a surface, or biofilms, represent the major lifestyle of bacteria in the environment. Such a sessile state enables the inhabitants to be more resistant to adverse environmental conditions. Thus, having a deeper understanding of the underlying mechanisms that regulate the transition between the motile and the sessile states could help design strategies to improve biofilms when they are beneficial or impede them when they are detrimental. For *Caulobacter crescentus* motile cells, the transition to the sessile lifestyle is irreversible, and this decision is regulated at several levels. In this work, we describe a putative phosphorelay that promotes the motile lifestyle and inhibits biofilm formation, providing new insights into the control of adhesin production that leads to the formation of biofilms.

## INTRODUCTION

In the environment, bacteria live primarily as surface-associated colonies (1–3). Biofilms typically provide strategic advantages to their inhabitants. Indeed, cells in a biofilm can acquire transmissible DNA more easily and can be more resistant not only to xenobiotic compounds and changes in environmental conditions, but also to grazing by some planktonic feeding predators and phagocytosis by the host immune system (4). While most studied biofilm-forming bacteria rely on a complex extracellular matrix composed of proteins, polysaccharides, DNA, and other macromolecules to tightly adhere to the surface (5), many Alphaproteobacteria use a strong polar adhesin to irreversibly attach to surfaces and form biofilms (6, 7). The most extensively studied example of this type of adhesin is the *Caulobacter crescentus* holdfast.

**Fig 1:**
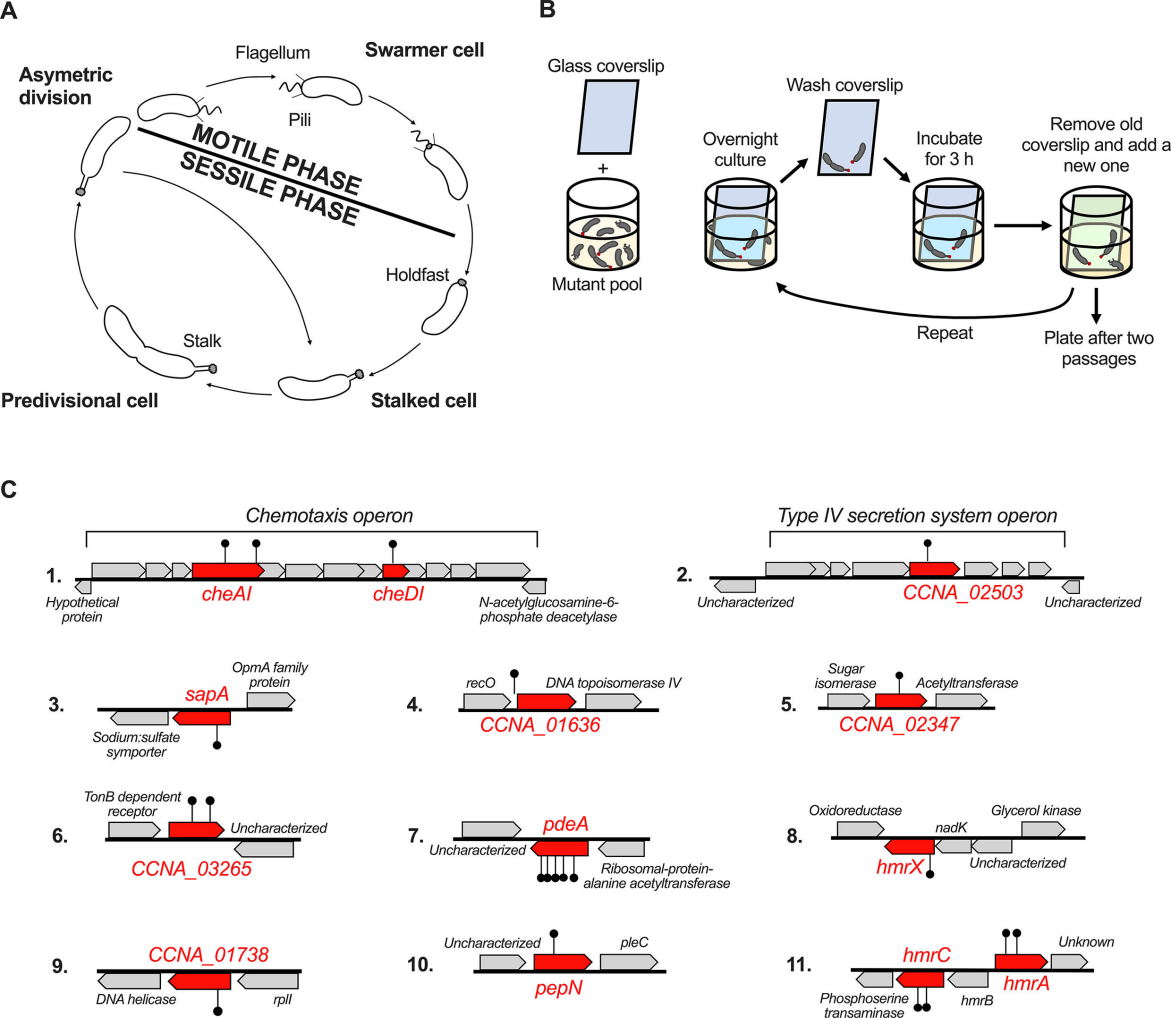
Adhesion enrichment screen for identification of hyperadhesive mutants. (**A**) Asymmetric cell cycle of *Caulobacter crescentus.* The newborn swarmer cell harbors pili and a flagellum at one pole of the cell. During the motile to sessile transition, this swarmer cell differentiates by retracting pili, ejecting the flagellum, and synthesizing a holdfast and a stalk at the same pole, giving rise to a stalked cell. This non-motile cell progresses through the cell cycle to give an elongated predivisional cell in which a new flagellum is synthesized at the pole opposite the stalked pole. After cell division, the newborn swarmer cell disperses and the stalked cell initiates the next round of replication. (**B**) Schematic of the forward genetic screen to identify mutants with an enhanced adhesion phenotype. A clean glass coverslip (depicted in blue) was added to a culture of pooled *Mariner* transposon mutants grown in M2X medium. After overnight growth, this coverslip was removed and thoroughly rinsed with sterile M2X, then used to inoculate a new culture. After 3 h of inoculation, the old coverslip (blue) was replaced with a new one (green). The enrichment process was repeated once more before plating. Individual mutants were isolated from single colonies. (**C**) Identity and location of the genes identified in the adhesion enrichment screen. Genes identified in this screen are indicated in red. The locations of the *Mariner* transposon insertions are indicated by black lollypops. The predicted function of the proteins encoded by the flanking genes is indicated in italics. For more information about the proteins encoded by the genes indicated in red, see Table 1.

*C. crescentus* cells have a dimorphic lifecycle, where each division cycle yields a sessile mother stalked cell and a motile daughter swarmer cell (Fig. 1A). Swarmer cells display a flagellum and multiple pili at one pole. During the transition to the sessile form, the flagellum is ejected, the pili are retracted, and cells enter the sessile phase of their life cycle. At the pole that was previously bearing the flagellum and pili, cells first synthesize a holdfast, followed by a cylindrical extension of the cell envelope called the stalk, which pushes the holdfast away from the cell body. The resulting stalked cells are attached to surfaces to form biofilms. Stalked cells eventually elongate, become predivisional cells, and synthesize a new flagellum at the pole opposite the stalk. After cytokinesis, each newborn swarmer cell disperses and must transition from its motile state to a stalked cell prior to the initiation of DNA replication and a new round of cell division.

The progression of the cell cycle and transition between motile and sessile phases are tightly regulated. Holdfast production is temporally regulated during the cell cycle, with genes involved in holdfast synthesis under the control of key cell cycle regulators (8, 9). The intracellular concentration of cyclic di-GMP (c-di-GMP) is the primary developmental regulator of the motile to sessile lifestyle transition (10) and is involved in both flagellar ejection and holdfast production (11–13). In addition to this internal signal, holdfast production is also controlled by external environmental signals such as nutrient availability, light, and general stress responses (14–17). These signals regulate the holdfast inhibitor HfiA, which inhibits the holdfast synthesis protein HfsJ, a predicted glycolipid glycosyltransferase crucial for holdfast formation (15). Regulation of HfiA is controlled using different mechanisms at the transcriptional and post-translational levels, including by c-di-GMP, enabling proper timing of holdfast synthesis (15, 18–20).

To better understand the underlying mechanisms that govern the motile to sessile lifestyle transition and holdfast regulation, we performed a genetic screen for mutations that enhance biofilm formation. This work reports a putative phosphorelay that inhibits the sessile lifestyle, while promoting motility. Phosphorelays, a sub-class of two-component systems (TCSs), are major regulatory pathways used by bacteria to sense and transduce environmental signals to trigger an adapted phenotypic response. A canonical TCS consists of a histidine kinase (HK)/response regulator (RR) pair. Hybrid histidine kinases (HHKs) are non-canonical TCSs, where the HK and the receiver domain of the RR are fused. HHKs represent less than 20% of bacterial HKs (21). Signal transduction from the HHK to the final RR typically involves a histidine phosphotransferase (HPT) as a mediator. Sixty-one percent of RR output domains contain a DNA-binding domain and regulate transcription (22). In addition, some output domains can transduce signals through diguanylate cyclase or phosphodiesterase domains, which produce and degrade c-di-GMP, respectively. This example highlights how regulation through phosphotransfer and c-di-GMP can be intertwined.

In this work, we describe a putative phosphorelay centered on the holdfast motility regulator A (HmrA), an HHK encoded by the *CCNA_03326* gene, identified in a screen for hyper-adhesive mutants. We show that this gene is involved in the switch from a motile to a sessile lifestyle. By controlling both the proportion of swarmer cells that synthesize a flagellum and the proportion of differentiating swarmer cells that synthesize a holdfast, it enables the fine-tuning of the relative number of cells harboring a flagellum or a holdfast in a mixed population. Our data suggest that HmrA regulation of holdfast production is linked to c-di-GMP production by DgcB. In addition, we identify two putative HPTs involved in the same pathway as HmrA, namely HmrB and HmrX. We also show that holdfast production is regulated by environmental signals such as the presence of excess Cu and different growth temperatures. This regulatory mechanism involves the protein HmrC, which is a predicted transmembrane protein required for sensing environmental stimuli that initiate the Hmr regulation cascade. Finally, our work provides evidence that this mechanism functions without modulating the transcription of the master regulator of holdfast synthesis, HfiA.

## RESULTS

### An enrichment for hyper-adhesive mutants identifies putative elements of phosphorelays

We sought to identify mutants with an enhanced adhesion phenotype. To sensitize our screen, cells were grown in defined M2X medium in which holdfast synthesis is significantly reduced compared to using the complex medium PYE (15, 16). We randomly mutagenized a pool of *C. crescentus* NA1000 *hfsA*^+^ wild-type (WT) using a *Mariner* transposon (23). Then, as depicted in Fig. 1B, transposon mutagenized cells were pooled, grown in the presence of a glass coverslip, and allowed to form biofilms. Cells adhering to the coverslip were recovered and enriched for two cycles. The adherent cell lines were isolated and the location of transposon insertions was determined. The enrichment resulted in 21 unique *Mariner* insertions that were mapped to 13 different genes (Table 1; Fig. 1C). One-third of the unique insertions were in genes encoding components of putative phosphorelays. Indeed, out of 21 hits, 4 occurred in putative HHK genes (*CCNA_03265* and *CCNA_03326*), 1 was in a putative HPT gene (*CCNA_01278*), and 2 in a gene of unknown function (*CCNA_03324*) located in the same gene cluster as the HHK *CCNA_03326*. We decided to focus on *CCNA_03326* and *CCNA_03324* since their presence in adjacent and divergent operons suggested that they might be involved in related functions. Furthermore, analysis of the gene neighborhood of *CCNA_03326,* using the Joint Genome Initiative server (24), revealed that this cluster is present only in representatives of the family of Caulobacteraceae. As described below, *CCNA_01278*, *CCNA_03324*, *CCNA_03325*, and *CCNA_03326* encode proteins involved in the regulation of holdfast synthesis and motility. We named these genes *hmrX*, *hmrC*, *hmrB*, and *hmrA*, respectively (for holdfast and motility regulators X, C, B, and A) (Fig. 1C).

**TABLE 1:** Hyperadhesive mutants obtained from the adhesion enrichment screen

Gene locus (CCNA)	Gene locus (CC)	Gene name	Predicted function	Unique Tn insertions
*CCNA_00442*	*CC_0433*	*cheAI*	Histidine kinase	2
*CCNA_00447*	*CC_0438*	*cheD*	Glutamine deamidase	1
*CCNA_00783*	*CC_0746*	*sapA*	Alkaline metalloproteinase	1
*CCNA_01278*	*CC_1220*	*hmrX*	Histidine phosphotransfer protein	1
*CCNA_01636*	*CC_1565*	N/A^*a*^	Alkaline phosphatase D	1
*CCNA_01738*	*CC_1666*	N/A	TonB-dependent receptor	1
*CCNA_02347*	*CC_2264*	N/A	Phosphoglucomutase	1
*CCNA_02503*	*CC_2421*	N/A	Type IV secretion	1
*CCNA_02566*	*CC_2481*	*pepN*	Aminopeptidase	1
*CCNA_03265*	*CC_3162*	N/A	Hybrid histidine kinase	2
*CCNA_03324*	*CC_3217*	*hmrC*	Unknown	2
*CCNA_03326*	*CC_3219*	*hmrA*	Hybrid histidine kinase	2
*CCNA_03507*	*CC_3396*	*pdeA*	Phosphodiesterase	5

^*a*^ N/A: not available.

**Fig 2:**
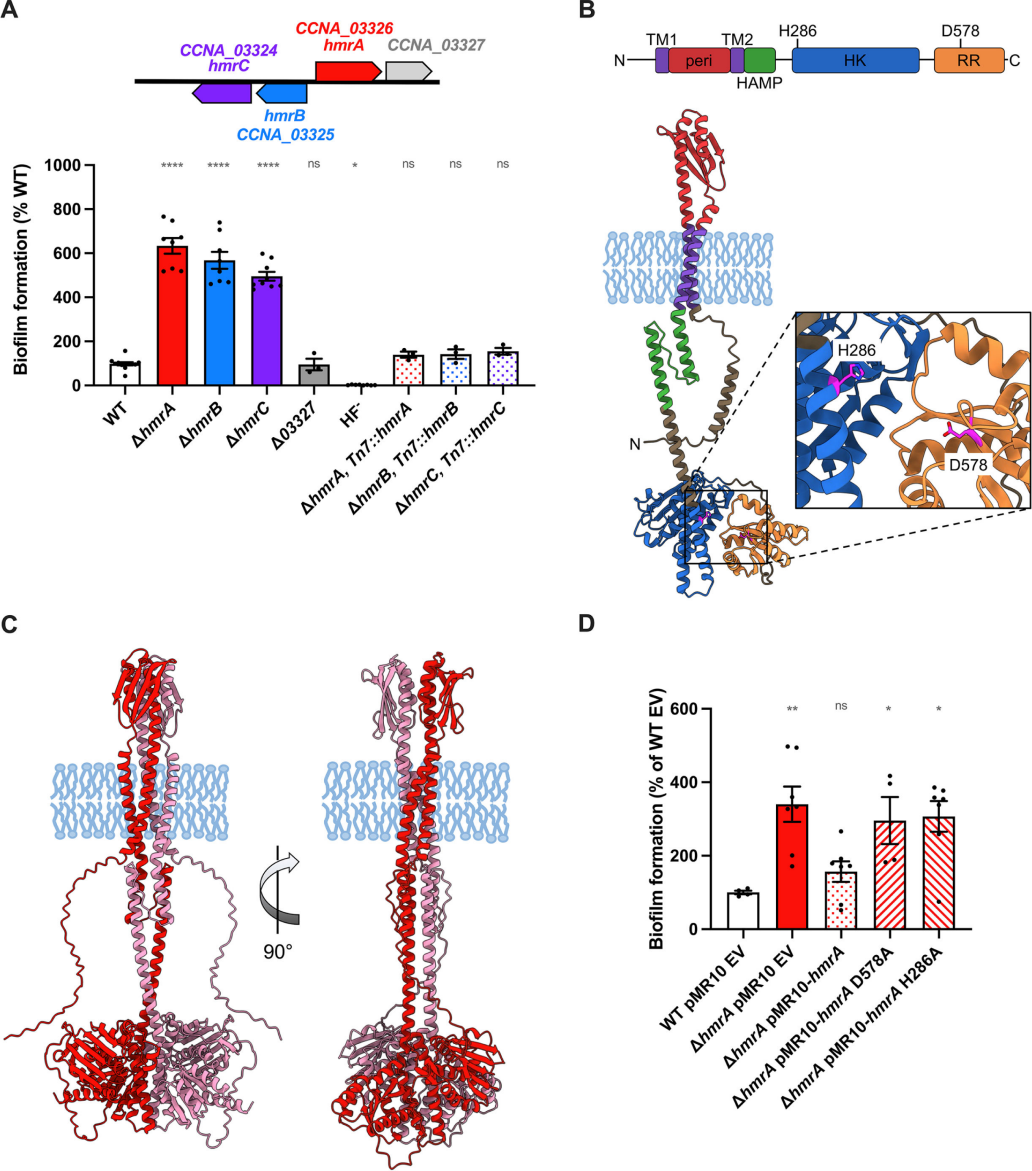
The putative HHK mutant ∆*hmrA* displays a hyperbiofilm phenotype. (**A**) Schematics of the *CCNA_03324-CCNA_03327* gene cluster (top) and biofilm formation (bottom) in 24-multiwell plates, after 24 h of incubation in M2X medium. Results are expressed as a percentage of biofilm formed by each strain compared to WT set at 100%. A holdfast-minus mutant HF^−^ (NA1000) was used as a no-adhesion negative control. (**B**) Predicted structure of HmrA. Top: The predicted domains are depicted as follows: purple: transmembrane (TM) regions, red: periplasmic (peri) domain, green: HAMP domain, blue: histidine kinase (HK) domain, and orange: response regulator (RR) domain. The putative sites of phosphorylation, H286 and D578, are indicated. Bottom: HmrA structure as predicted using AlphaFold (AFDB A0A0H3CC19), with domains of interest colored as described above. (**C**) HmrA dimer structures predicted by AlphaFold-multimer as implemented within ColabFold. Two monomers of HmrA are shown in red and pink, respectively. (**D**) Biofilm formation in 24-multiwell plates, after 24 h of incubation in M2X medium. Results are expressed as a percentage of biofilm formed by each strain compared to WT bearing the empty pMR10 vector (EV) set at 100%. All biofilm results are given as the average of at least three independent experiments, each run in triplicate, and the error bars represent the SEM. Statistical significance comparisons to WT (100%) are calculated using one-sample *t* tests to determine if the mean of each sample differs significantly from 100. ns: not significant; *P < 0.05 ; **P < 0.01; ****<*P* < 0.0001.

A mutant strain harboring an in-frame deletion of the *hmrA* gene was generated to eliminate the potential polar effect of a transposon insertion. The ∆*hmrA* mutant exhibited a hyper-adhesive phenotype, with approximately sevenfold more biofilm formed than the WT strain after 24 h of incubation (Fig. 2A). This indicates that the putative HHK encoded by *hmrA* inhibits biofilm formation. We also constructed in-frame deletions of the other ORFs in the *hmr* gene cluster (Fig. 1C and 2A) and tested these mutants for their ability to form biofilms. The behaviors of both ∆*hmrC* and ∆*hmrB* were very similar to that of ∆*hmrA* (Fig. 2A). In contrast, ∆*CCNA_03327* phenocopied the WT (Fig. 2A). We complemented each mutant strain by inserting a single copy of the deleted gene with its native promoter at the Tn*7 att* site, resulting in near WT biofilm levels for each of the complemented strains (Fig. 2A). To determine if the Hmr-mediated hyper-adhesive phenotype was restricted to defined M2X medium, we also performed biofilm experiments in complex PYE medium (Fig. S1). Our results show that ∆*hmrA*, ∆*hmrB*, and ∆*hmrC* also form more biofilm than WT in PYE, indicating that the observed hyperadhesive phenotype is not specific to the metabolic constraints of the defined medium M2X.

**Fig 3:**
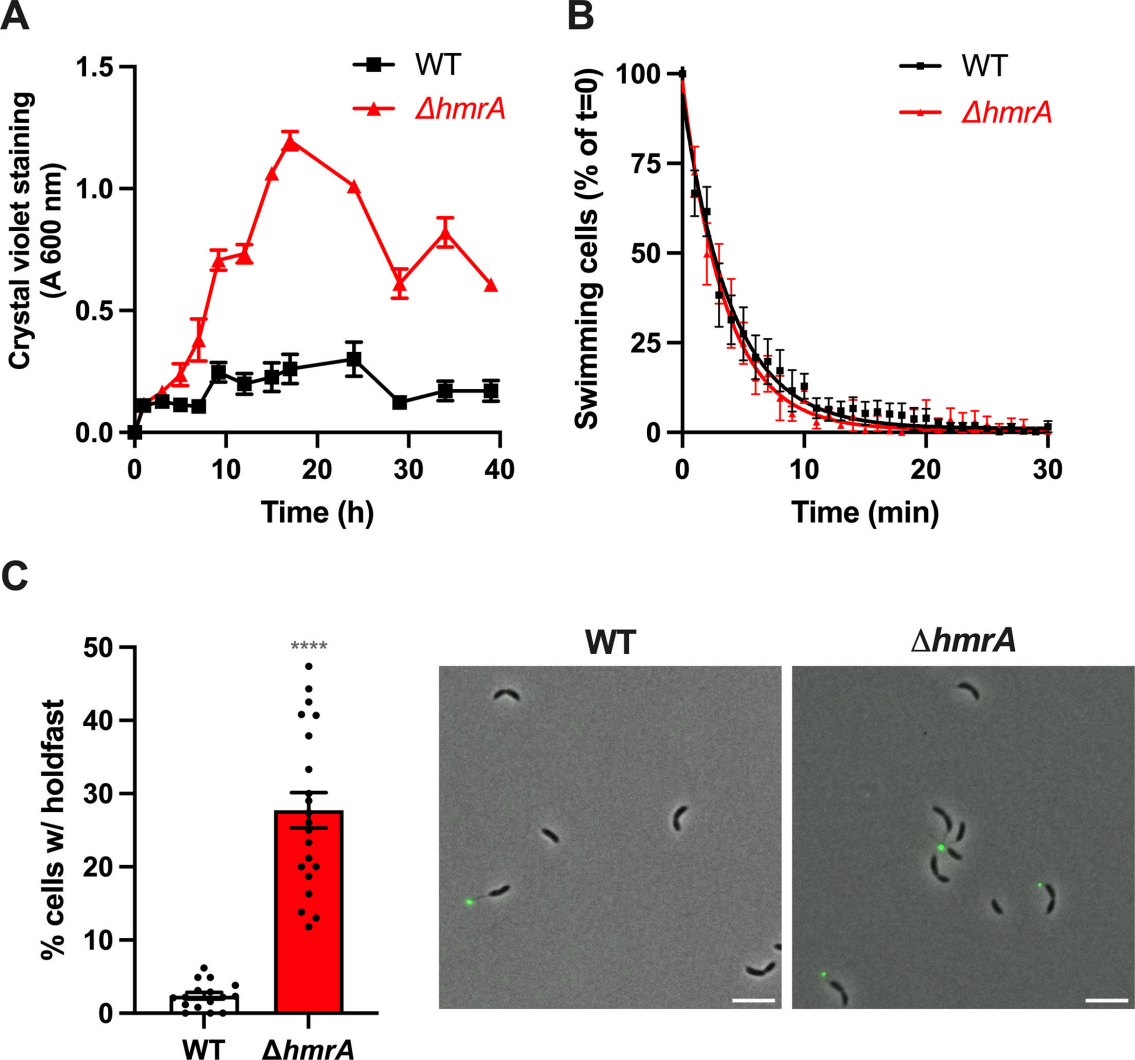
The ∆*hmrA* mutant produces more biofilm and holdfasts. (**A**) Biofilm formation over time. Amount of biofilm formed by WT (black squares) and ∆*hmrA* (red triangles) over time in M2X medium. Cultures were grown in 24-multiwell plates and the amount of biomass attached to the inside of the wells over time was quantified using crystal violet. Data are given as the average of crystal violet staining of three independent experiments with SEM. (**B**) Single-cell adhesion of WT (black) and *∆hmrA* (red) synchronized swarmer cells to a glass surface. The number of motile cells per field of view for different time points is shown as a percentage of swimming cells compared to the beginning of the experiment (*t* = 0; 100%) and the results are averages from five samples and three independent experiments. Data were fitted using an exponential curve and error bars represent the SEM. (**C**) Left: quantification of cells harboring a holdfast. Holdfasts from mixed populations of WT and ∆*hmrA* grown to mid-exponential phase were labeled using AF488-WGA, and the number of cells harboring a holdfast per field of view was quantified by microscopy. The results are given as the percentage of cells fluorescently labeled (bearing a holdfast) over the entire population and represent the average of at least three independent replicates (more than 300 cells per replicate) with SEM. The statistical comparison is calculated using an unpaired *t* test (****P < 0.0001). Right: representative images of cells grown to mid-exponential phase in M2X and labeled with AF488-WGA to visualize holdfasts (scale bar = 2 µm).

### HmrA is an HHK involved in biofilm regulation

The amino acid sequence of HmrA was analyzed using the conserved domain architecture database (CDART) and ExPasy Prosite (25, 26). HmrA displays typical HK and RR domains (Fig. 2B). Moreover, a HAMP linker domain was identified between amino acids (aa) 201 and 254. Such a domain is typically found in transmembrane histidine kinases where it facilitates signal transduction between a periplasmic domain and the HK domain (27). In addition, two transmembrane helices flanking a periplasmic domain, between residues 71 and 177, were predicted using the DeepTMHMM server (28) (Fig. 2B). To get a better sense of the domain architecture, the AlphaFold2 (AF2)-predicted structure of HmrA was obtained from the AlphaFold Protein Structure Database (AFDB A0A0H3CC19) (29) and the abovementioned domains mapped onto the structure. The sequence-based domain predictions were in good agreement with the AF2-derived structure. Analysis of the predicted HmrA structure using Foldseek (30) suggested that the cytosolic region of HmrA most closely resembles the *C. crescentus* HHK ShkA (PDB 6QRJ), while the periplasmic region of HmrA most closely resembles an ortholog of the *Pseudomonas fluorescens* LapD periplasmic output domain (PDB 4U65). Since both ShkA and LapD adopt dimeric arrangements, and HK and HAMP domains often form homodimers generally (31), we utilized AlphaFold-multimer (AF-multimer) (32) as implemented in ColabFold (33) to predict whether HmrA may similarly form a homodimer (Fig. 2C; Fig. S2). Indeed, AF-multimer generated very consistent and confidently predicted models of a HmrA dimer whose interaction interface spans the periplasmic, transmembrane, and cytosolic regions of the protein (Fig. S2), as has been observed for other experimentally determined structures of HKs (34). Thus, HmrA is likely to be a transmembrane HHK that can transduce a signal from the periplasm to the cytoplasm.

The Molecular Signal Transduction database 3.0 (MiST 3.0) (35) reports 28 HHKs in the *C. crescentus* NA1000 genome. Alignment of the HmrA sequence with all 28 of these HHKs facilitated the identification of the putative catalytic histidine and aspartate residues (Fig. 2B ; Fig. S3). The HK and RR domains are generally well-conserved among these 28 HHKs, with H286 being the best candidate for the catalytic histidine and D578 for the catalytic aspartate in HmrA. The ∆*hmrA* mutant was transformed with a low-copy replicating plasmid bearing various alleles of *hmrA*, either wild-type or harboring alanine substitutions at H286 and D578. Expression of the original *hmrA* gene in the ∆*hmrA* background reverted the phenotype to WT, while *hmrA* with H286A or D578A substitutions did not complement (Fig. 2D). In addition, a previous study showed that HmrA exhibits *in vitro* kinase activity (36). Taken together, these data strongly suggest that HmrA acts as an HHK.

### HmrA regulates holdfast synthesis

To better assess the hyper-biofilm phenotype of the ∆*hmrA* mutant, we monitored the adhesion of both WT and ∆*hmrA* strains over time. For the first few hours, WT and ∆*hmrA* strains adhered to the surface in a similar manner (Fig. 3A). However, after 5 h post-inoculation, ∆*hmrA* cells started to exhibit an enhanced adhesion phenotype that continued to increase over time (Fig. 3A). In parallel, we monitored initial adhesion events at the single cell level by recording the binding of synchronized swarmer cells to a glass surface over time by microscopy (Fig. 3B). We found that the percentage of swimming cells decreased at the same rate for the WT and ∆*hmrA* strains (Fig. 3B). This showed that the ability of single motile cells to interact with the surface is not impacted in the ∆*hmrA* mutant.

In *C. crescentus*, biofilm formation and permanent adhesion require the adhesive holdfast (7). Therefore, to test whether the enhanced adhesion phenotype of ∆*hmrA* could be due to the misregulation of holdfast synthesis, we first quantified the number of cells harboring a holdfast in mixed populations of cells for both WT and ∆*hmrA* strains (Fig. 3C). We found that approximately 2.5% of total WT cells harbored a holdfast, while this percentage reached more than 25% for the ∆*hmrA* mutant. When cells are grown in complex medium, holdfast synthesis is triggered within seconds upon contact with the surface, bypassing the developmental pathway (7, 37). However, it has been shown previously that holdfast production upon surface contact does not occur when cells are grown in defined media such as M2X (38). Therefore, since the increase in cells harboring a holdfast in a mixed ∆*hmrA* population occurs in defined M2X medium, this phenotype is independent of surface contact stimulation of holdfast synthesis.

To investigate if HmrA is involved in the developmental pathway of holdfast synthesis, we monitored the timing of holdfast synthesis of newborn cells during the cell cycle by time-lapse microscopy (Fig. S4A). The duration of a complete cell cycle was identical for both WT and ∆*hmrA* strains (Fig. S4B). Within that cell cycle, holdfast synthesis occurred at the same time, after approximately 30 min in both strains (Fig. S4C).

Taken together, these results suggest that HmrA is not involved in regulating the cell cycle timing of holdfast synthesis at the single cell level, but rather determines the proportion of cells that synthesize a holdfast.

### HmrA affects the proportion of motile cells in culture

**Fig 4:**
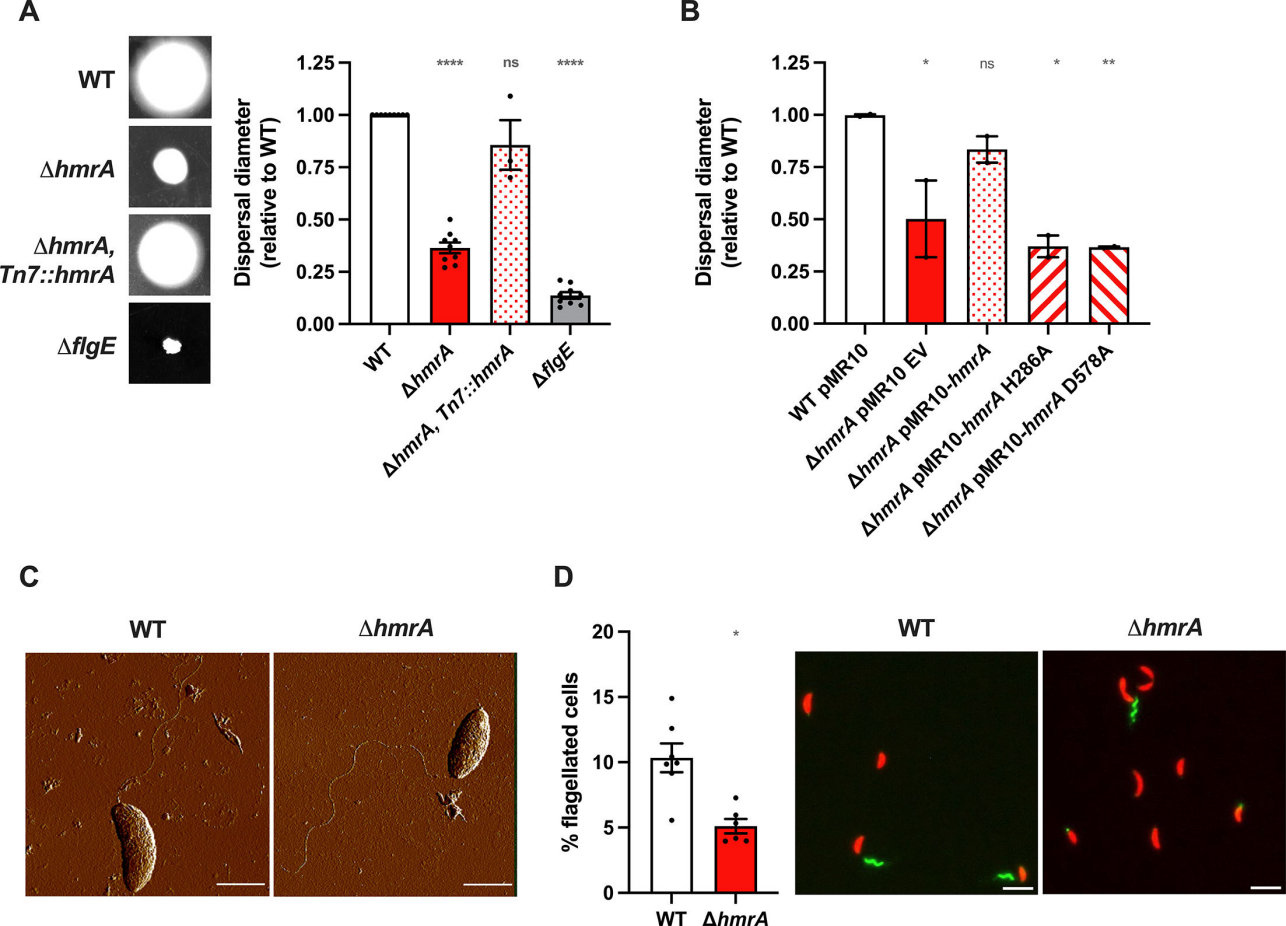
The *∆hmrA* mutant is impaired for swimming through semisolid medium because of a lower proportion of flagellated cells. (**A**) Motility assays in semisolid agar for WT and *∆hmrA* strains, using M2X medium + 0.4% noble agar. Representative images of swim rings obtained after 5 days of incubation in M2X semisolid plates are shown on the left. On the right, dispersal diameters of various strains are shown normalized to WT ring diameter measured on the same plate (set to 1). A non-motile strain lacking its flagellum (NA1000 *hfsA*^+^ ∆*flgE*) was used as a non-swimming negative control. (**B**) Motility assays through semisolid agar for the ∆*hmrA* point mutants. Results are normalized to the swim ring diameter of WT bearing the empty pMR10 vector and measured on the same plate (set to 1). Bar graphs indicate the mean of three independent replicates, and error bars represent SEM. Statistical comparisons to WT or WT pMR10 are calculated using one-sample *t* tests to determine if the mean of each sample differs significantly from 1. (**C**) High-resolution AFM images showing a WT and ∆*hmrA* swarmer cell harboring a flagellum located at the pole (scale bar = 1 µm)**.** (**D**) Quantification of the total number of flagella detected in mixed populations. Cells were grown to OD_600_ = 0.3–0.5 and the number of cells bearing a flagellum was scored using microscopy images where cells express dsRed (red cell bodies) and flagella are labeled using AF488-maleimide dye (green filaments). The results represent the average of at least three independent replicates (more than 500 cells per replicate) and the error bars represent the SEM. Statistical comparison is calculated using an unpaired *t* test. Representative images are shown on the left (scale bar = 2 µm). ns: not significant; *P < 0.05; **P < 0.01; ****P < 0.0001.

In *C. crescentus*, holdfast misregulation is often linked to changes in motility (16, 38, 39), so we investigated the role of HmrA in motility. First, we monitored collective motility (at the population level) by measuring the swimming-dependent dispersal behavior of WT and ∆*hmrA* strains through semisolid agar M2X plates. Compared to WT, ∆*hmrA* displayed greatly reduced swimming dispersal under these conditions: the dispersal diameter of a ∆*hmrA* strain was only a third of WT (Fig. 4A). This defective swimming dispersal phenotype could be reversed by complementing ∆*hmrA* with a single copy of *hmrA* under the control of its native promoter at the Tn*7 att* site (Fig. 4A). This result suggests that HmrA promotes swimming motility. Residues H286 and D578 in HmrA, which we identified previously as crucial for regulating biofilm formation (Fig. 2D), were equally important to promote dispersal through semisolid agar (Fig. 4B). We performed a similar experiment using complex PYE medium (Fig. S1) and also observed smaller dispersal rings for the ∆*hmrA* mutant compared to WT, indicating that nutrient availability is not responsible for this phenotype.

A collective swimming defect through semisolid agar can be the consequence of various causes. We first checked if this phenotype was linked to the increased holdfast synthesis reported above. Indeed, mis-timed holdfast synthesis during the swarmer cell phase can result in a loss of motility and premature adherence to surfaces (15, 40, 41). Therefore, we measured the ability of holdfast-minus (HF^−^) strains to disperse through semisolid agar. We showed that a holdfast defective NA1000 ∆*hmrA* (HF^−^) motility phenotype is identical to that of a holdfast harboring NA1000 *hfaA^+^* ∆*hmrA* mutant (Fig. S5A). Thus, we conclude that the observed impaired swimming dispersal phenotype of the ∆*hmrA* mutant through semisolid agar is not dependent on the presence of a holdfast.

We next tested whether the dispersal phenotype of the ∆*hmrA* mutant could result from a defect in chemotaxis, which can result in a decrease in the size of the dispersal ring (42, 43). In addition, it has been shown that CheA and CheB chemotaxis proteins regulate both chemotaxis and holdfast production in *C. crescentus* (16), and that the CheY-like c-di-GMP effector proteins CleA and CleD also play a role in the regulation of holdfast synthesis (44). Altogether, this shows that chemotaxis, dispersal through semisolid agar, and holdfast production can be linked. To test chemotaxis in the ∆*hmrA* mutant, the cells were placed on a plate where xylose was introduced at the center of the plate as the sole carbon source and chemoattractant (45, 46), diffusing outward to create a gradient. Cells with a functional chemotaxis system preferentially swam toward the xylose, creating an asymmetric ring skewed toward the higher carbon concentrations (Fig. S5B). WT, ∆*hmrA*, and ∆*cheAI* (a negative control that cannot perform chemotaxis [16, 47]) strains were tested and the plates were analyzed after 48 h (Fig. S5B). As expected, the ∆*cheAI* strain produced an evenly distributed ring, showing that it is unable to respond to the xylose gradient and to display a chemotaxis phenotype. In contrast, WT and ∆*hmrA* both produced asymmetric rings toward the carbon source, showing that chemotaxis is functional in these strains. Due to its previously mentioned impaired capability in swimming dispersal, the ∆*hmrA* mutant produced a less pronounced signal than WT. Nevertheless, asymmetry of its growth ring confirmed that its chemotaxis is functional.

Finally, another cause of dispersal impairment through semisolid agar can be a defect in swimming behavior. Therefore, we measured the swimming speed of synchronized motile swarmer cells in liquid M2X medium and determined that single cells of WT and ∆*hmrA* display similar distributions of swimming speed (Fig. S5C). Furthermore, flagellum localization and structure were not qualitatively different, as observed by atomic force microscopy (AFM) (Fig. 4C). However, when we quantified the number of cells with a flagellum in a mixed population, we found that the proportion of ∆*hmrA* cells harboring a flagellum is approximately half of WT total cells (Fig. 4D). Among these flagellated cells, the proportion of swarmer and predivisional cells harboring a flagellum was similar for both WT and the ∆*hmrA* strain (Fig. S5D). These results indicate that while the ∆*hmrA* mutation reduces the number of cells with a flagellum in culture, it does so similarly in swarmer and predivisional cells. This is consistent with the fact that the cell cycle is not perturbed, as shown above (Fig. S4).

Taken together, these results show that, while each ∆*hmrA* cell that harbors a flagellum behaves like WT, the overall proportion of flagellated cells is lower in the ∆*hmrA* population, explaining the decreased collective dispersal efficiency through semisolid agar. Based on these experiments, we suggest that HmrA regulates the balance between the number of flagellated cells and the number of cells harboring a holdfast in the population.

### HmrA does not impact cell cycle progression

We showed above that HmrA regulates neither the timing of holdfast synthesis at the single cell level (Fig. S4), nor the proportion of swarmer and predivisional cells harboring a flagellum (Fig. S5D). These results suggest that HmrA does not play a role in overall cell cycle regulation. To further confirm that the observed differences in flagellum and holdfast formation of the ∆*hmrA* mutant are not due to a misregulation of cell cycle progression, we investigated major key events in cell differentiation. First, as mentioned above, time-lapse experiments showed that the timing of the overall cell cycle is identical in ∆*hmrA* and WT (Fig. S4). In addition, chromosome replication and partitioning were similar in WT and ∆*hmrA* (Fig. S6A through D), as was the timing of swarmer to stalked cell differentiation assessed by determining the timing of PleC delocalization and DivJ localization (Fig. S6E and F). In aggregate, these results show that there is no difference in cell cycle timing and cell differentiation between WT and ∆*hmrA.*

In addition, previous studies showed that sensing of a surface by pili triggers premature cell differentiation and holdfast production in *C. crescentus* (40, 41). We therefore assessed pilus formation in ∆*hmrA* and saw no noticeable difference between WT and ∆*hmrA*. Both strains were similarly sensitive to infection with phage ΦCbK (Fig. S7A), suggesting that ∆*hmrA* produces functional pili. Moreover, the slight increase in dispersion of the ∆*pilA* mutant compared to WT is recapitulated in the ∆*hmrA* ∆*pilA* mutant compared to the ∆*hmrA* strain (Fig. S7B). Deletion of *hmrA* in the WT or ∆*pilA* background improved biofilm formation similarly by 6- to 8-fold compared to the parent strains (Fig. S7C). Finally, the proportion of cells bearing a holdfast in a mixed population was increased by 6 to 7.5-fold when *hmrA* was deleted in either the WT or ∆*pilA* background (Fig. S7D). These experiments strongly suggest that the ∆*hmrA* phenotype is not due to changes in pilus regulation.

Taken together, the above data show that HmrA does not control pilus formation or activity and does not regulate flagellum or holdfast formation via cell cycle timing.

### Suppressors of the ∆*hmrA* motility defect map to genes involved in c-di-GMP modulation

**Fig 5:**
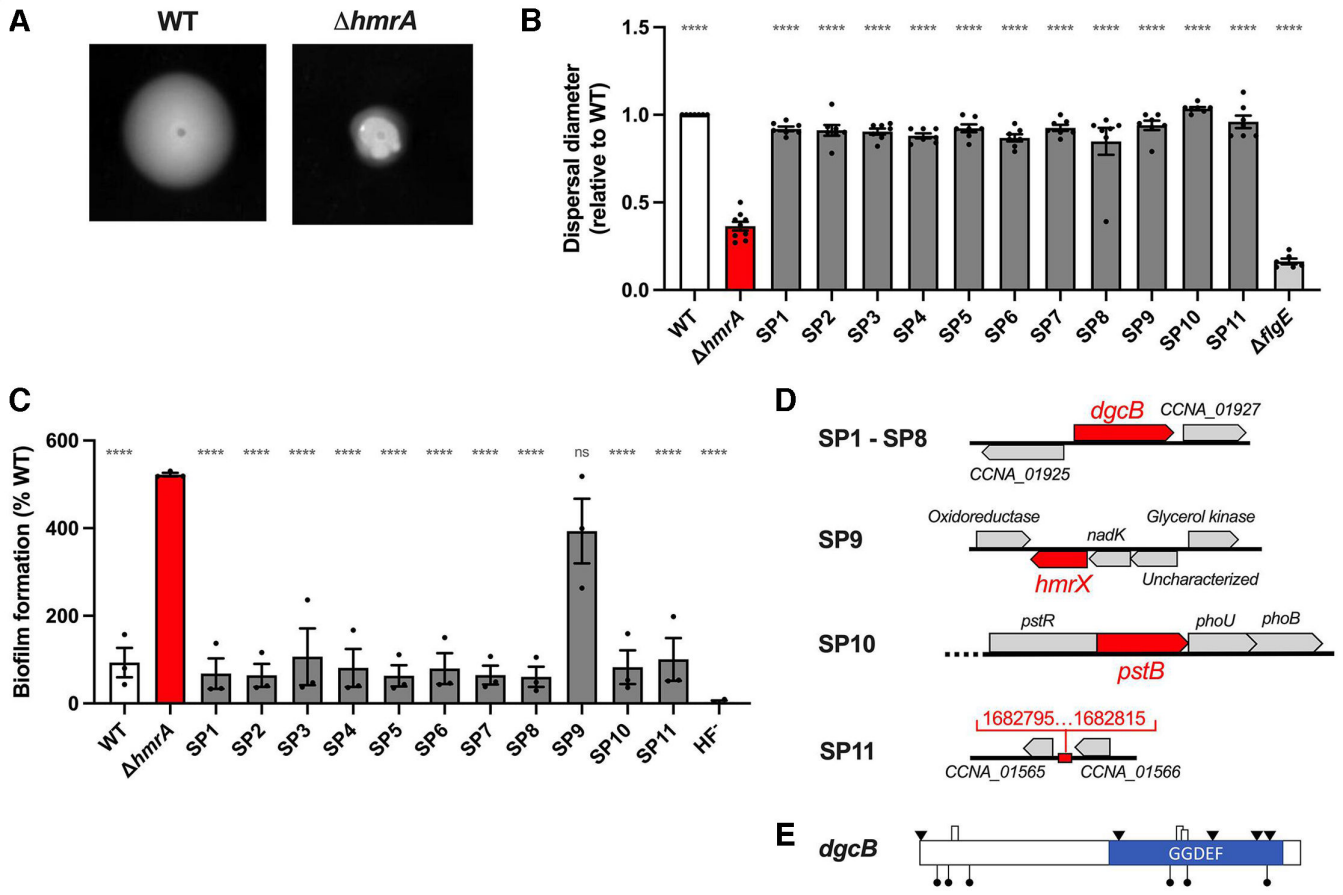
Identification of mutants that suppress the motility deficiency of ∆*hmrA* in semisolid agar plates. (**A**) Representative examples of semisolid agar plates inoculated with WT (left) and the ∆*hmrA* mutant (right). Both strains were inoculated into a PYE agar plate containing 0.4% noble agar and imaged after 7 days at room temperature. Flares of spontaneous suppressors in the ∆*hmrA* background are visible on the right panel. (**B**) Motility assays through M2X semisolid agar. Swim rings obtained after 5 days of incubation at room temperature were measured and results normalized to WT ring diameter measured on the same plate (set to 1). Bar graphs indicate the mean of three independent replicates, and error bars represent SEM. (**C**) Biofilm formation in 24-multiwell plates after 24 h of incubation in M2X medium of the ∆*hmrA* suppressors. Results are the average of three independent experiments, each run in triplicate, and the error bars represent the SEM and are expressed as a percentage of biofilm formed by each strain compared to WT set at 100%. For panels **B** and **C**, statistical differences to ∆*hmrA* are calculated using ANOVA and a Benjamini-Hochberg protocol for multiple comparisons (ns: non-significant; ****P < 0.0001). (**D**) Genes that were mutated in the suppressor strains (red) and locus organization. (**E**) Schematic of the *dgcB* gene and location of the mutations found in the different suppressor strains (SP1 to SP8), with point mutations illustrated as black triangles and insertions/deletions as boxes. The locations of the *Mariner* transposon insertions are indicated by black lollypops. For more information about these mutations, see Table 2.

To gain more insight into the Hmr pathway, we performed a screen for spontaneous suppressors of the *hmrA* dispersal defect after noticing the frequent appearance of spontaneous suppressor flares through semisolid agar (Fig. 5A). Eleven of these flares (SP1 to SP11) were selected for further characterization and were tested for dispersal ability through semisolid agar and for biofilm formation (Fig. 5B and C). Their phenotypes were very similar to WT, with an increased dispersal through semisolid agar and decreased biofilm formation compared to the ∆*hmrA* parent strain. Only the SP9 suppressor displayed a level of biofilm formation that was similar to its parental strain ∆*hmrA*, while its dispersal through semisolid agar was similar to WT, like the rest of the suppressors. The location and nature of the different mutations are summarized in Table 2; Fig. 5D. Eight out of 11 spontaneous suppressors had distinct mutations in the diguanylate cyclase gene *dgcB* (*CCNA_01926*) (Table 2; Fig. 5D), a known regulator of holdfast production (13). DgcB is involved in the mechanosensing pathway of holdfast production through interaction with the flagellar motor to trigger the production of c-di-GMP, leading to the c-di-GMP-dependent activation of the holdfast synthesis protein HfsJ (19, 48).

**TABLE 2:** ∆*hmrA* suppressors displaying semisolid swimming phenotype

Suppressor name	Gene locus (CCNA)	Gene locus (CC)	Gene name	Mutation*^a^*	Frameshift mutation
SP1	*CCNA_01926*	*CC_1850*	*dgcB*	∆*G96-C115*	Yes
SP2	*CCNA_01926*	*CC_1850*	*dgcB*	∆*T740-G757*	No
SP3	*CCNA_01926*	*CC_1850*	*dgcB*	∆*C751-C768*	No
SP4	*CCNA_01926*	*CC_1850*	*dgcB*	*A573T*	No
SP5	*CCNA_01926*	*CC_1850*	*dgcB*	*G827:: ^b^*	Yes
SP6	*CCNA_01926*	*CC_1850*	*dgcB*	*T955C*	No
SP7	*CCNA_01926*	*CC_1850*	*dgcB*	*A986::A*	Yes
SP8	*CCNA_01926*	*CC_1850*	*dgcB*	*T1::A*	Yes
SP9	*CCNA_01278*	*CC_1220*	*hmrX*	A152T	No
SP10	*CCNA_00294*	*CC_0292*	*pstB*	T275P	No
SP11				*A1682805T^c^*	No

^a^ Mutations indicated in the mutation column were identified through sequencing of *dgcB* or the entire genome.

^b^ The * in SP5 represents the insertion of the following DNA sequence ACCACGCTGGAGGAAACG CGAAGCCGCCTCGGTGGTGGCG.

^c^ The mutation in SP11 mutant is in an intergenic region and the numeric site of the mutation is indicated.

**Fig 6:**
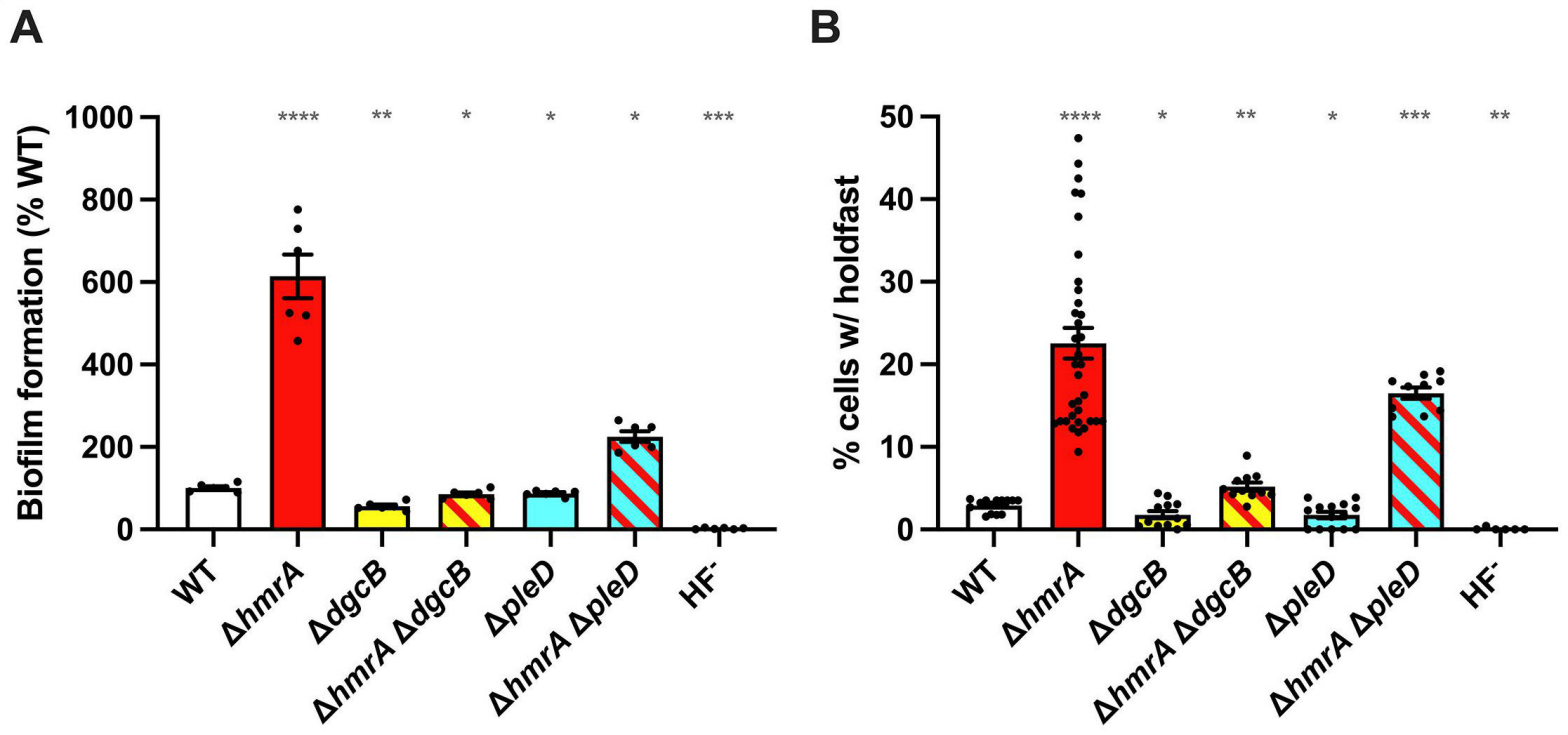
Adhesion phenotypes of the ∆*dgcB* and ∆*pleD* mutants. (**A**) Biofilm formation in 24-multiwell plates, after 24 h of incubation in M2X medium. Results are expressed as a percentage of biofilm formed by each strain compared to WT set at 100%. Results are given as the average of three independent experiments, each run in triplicate, and the error bars represent the SEM. (**B**) Quantification of cells harboring a holdfast. Holdfasts from mixed populations of each strain grown to mid-exponential phase were labeled using AF488-WGA, and the number of cells harboring a holdfast per field of view was quantified by microscopy. The results are given as the percentage of cells fluorescently labeled (bearing a holdfast) over the entire population, and represent the average of at least three independent replicates (more than 300 cells per replicate) and the error bars represent the SEM. For both graphs, statistical differences were calculated using one-sample *t* tests to determine if the mean of each sample differs significantly from 100 (**A**) or 1 (**B**) (*P < 0.05; **P < 0.01; ***P < 0.001; ****P < 0.0001).

In parallel, we also performed a similar suppressor screen by *Mariner* transposon mutagenesis and identified transposon insertions that restored dispersal to ∆*hmrA* through semisolid agar plates. All of the six analyzed mutants presented unique insertions in *dgcB* (Fig. 5E). Interestingly, *dgcB* was the only diguanylate cyclase gene identified in both ∆*hmrA* motility suppressor screens. It has been shown that the diguanylate cyclases PleD and DgcB are involved in flagellum and holdfast regulation via the developmental and mechanical pathway, respectively (48). Therefore, we constructed *dgcB* or *pleD* mutants in the ∆*hmrA* background to determine if HmrA is involved in the mechanical or developmental holdfast production pathway, or both, by monitoring biofilm formation (Fig. 6A) and number of cells harboring a holdfast (Fig. 6B). Deleting *dgcB* or *pleD* in a WT background decreased the number of cells harboring a holdfast by approximately 40% (2.9% ± 0.8%, 1.8% ± 1.6%, and 1.7% ± 1.5% of the number of cells harboring a holdfast for WT, ∆*dgcB,* and *∆pleD,* respectively). In contrast, deleting *hmrA* in a WT or ∆*pleD* background caused an increase in cells bearing a holdfast in the population by 8–10 times (22.6 ± 11 and 16.5 ± 2.2 for *∆hmrA* and *∆hmrA ∆pleD,* respectively). The ∆*hmrA* ∆*dgcB* mutant only produced around three times more holdfasts (5.2 ± 1.6%) than the ∆*dgcB* single mutant. Thus, while deleting *hmrA* in a *pleD* background phenocopies a *hmrA* deletion in WT, this is not the case in a *dgcB* background. These results strongly suggest that the *hmrA* phenotypes are specifically due to an increase in DgcB-dependent c-di-GMP synthesis and linked to the mechanical pathway of holdfast production (48). In support of this hypothesis, the aforementioned suppressor screens revealed DgcB as the sole putative diguanylate cyclase partner for HmrA.

### HmrX and HmrB are putative histidine-phosphotransferases working in concert with HmrA

Another spontaneous *hmrA* suppressor mutant that caught our attention is SP9 (Table 2). This mutant has an A152T mutation in HmrX. This point mutation restores the WT phenotype for swimming through semi-solid agar (Fig. 5B), but not for biofilm formation (Fig. 5C): this mutant still displays a biofilm phenotype similar to ∆*hmrA* (Fig. 5C). Interestingly, during our original screen for hyperadhesive mutants (Fig. 1B), a mutant with a *Mariner* transposon insertion in *hmrX* was also isolated (Fig. 1C). The finding of mutations in this gene in both screens highly suggests that HmrX is involved in the regulation of both adhesion and motility, like HmrA.

**Fig 7:**
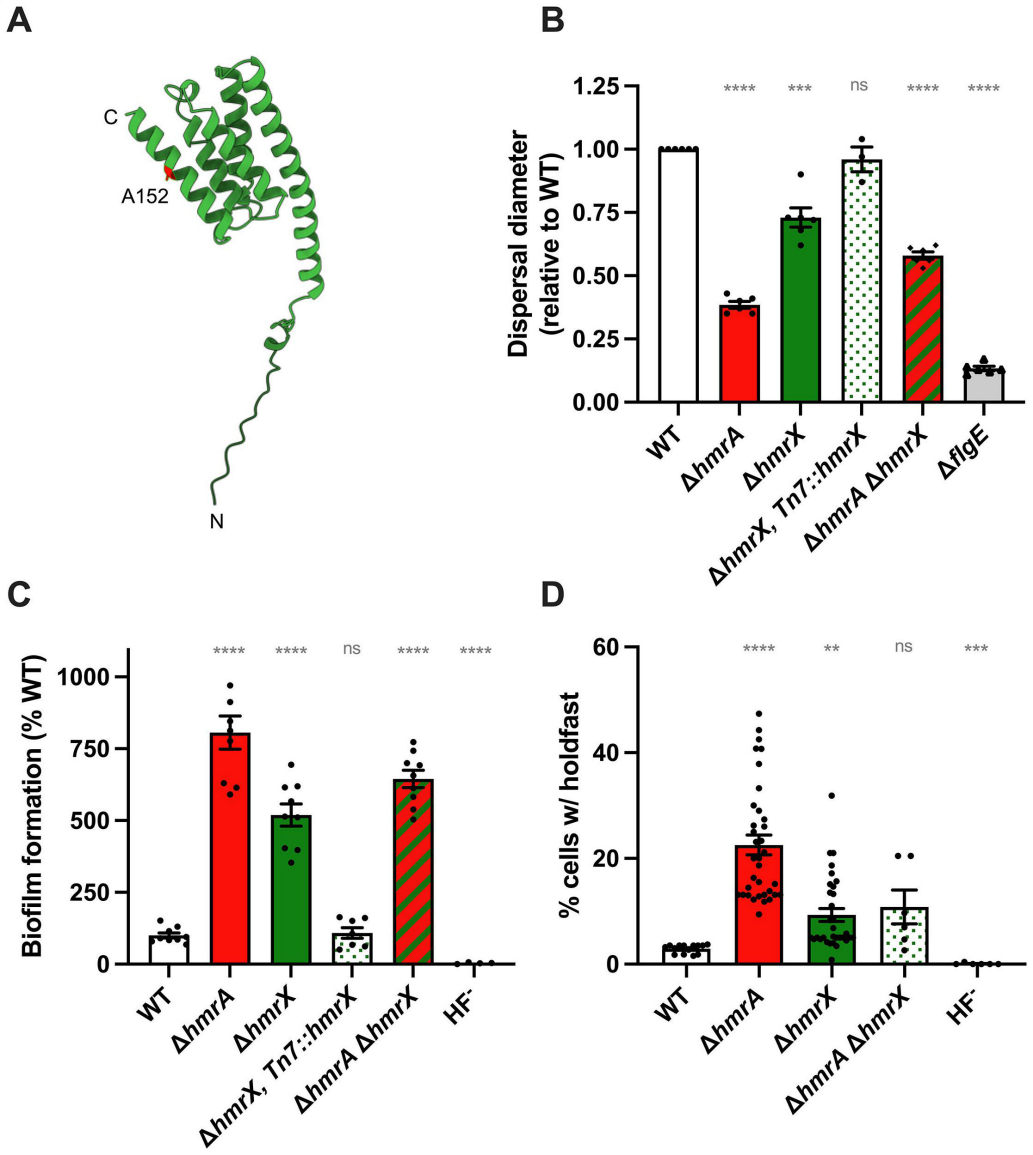
HmrX, a putative HTP involved in the Hmr pathway. (**A**) HmrX structure as predicted using AlphaFold (AFDB A0A0H3C6Y4); the A152 residue (substituted to a T in the SP9 suppressor mutant) is highlighted in red. (**B**) Motility assays through M2X semisolid agar. Swim rings obtained after 5 days of incubation at room temperature were measured and results were normalized to WT ring diameter measured on the same plate (set to 1). Bar graphs indicate the mean of three independent replicates with SEM. Statistical comparisons are calculated using one-sample *t* tests to determine if the mean of each sample differs significantly from 1. (**C**) Biofilm formation in 24-multiwell plates, after 24 h of incubation in M2X medium. Results are expressed as a percentage of biofilm formed by each strain compared to WT set at 100%. Results are given as the average of three independent experiments, each run in triplicate, and the error bars represent the SEM. Statistical comparisons are calculated using one-sample *t* tests to determine if the mean of each sample differs significantly from 100. (**D**) Quantification of cells harboring a holdfast. Holdfasts from mixed populations of each indicated strain grown to mid-exponential phase were labeled using AF488-WGA, and the number of cells harboring a holdfast per field of view was quantified by microscopy. The results are given as the percentage of cells fluorescently labeled (bearing a holdfast) over the entire population and represent the average of at least three independent replicates (more than 300 cells per replicate), with the error bars representing the SEM. Statistical comparisons to WT are calculated using ANOVA and a Benjamini-Hochberg protocol for multiple comparisons. ns: not significant; *P < 0.05; **P < 0.01; ***P < 0.001; ****P < 0.0001.

The AF2-predicted structure (AFDB A0A0H3C6Y4) suggests that HmrX is likely to adopt a four-helix bundle arrangement (Fig. 7A). The formation of a four-helix bundle is a feature of HTPs, which are otherwise poorly conserved in terms of primary sequence. The A152 residue is located in one of the helices of the four-helix bundle, with access to the surrounding solvent. Analysis of this structure using Foldseek suggests that HmrX most closely resembles the P1 domain of CheA orthologs, including those of *Escherichia coli* (PDB 8C5V), *Thermotoga maritima* (PDB 2LD6), and *Salmonella typhimurium* (PDB 1I5N). The P1 domain is the site of CheA autophosphorylation (49), supporting a role for HmrX as an HPT, as was previously suggested from an *in silico* search for HPTs in the *C. crescentus* genome (50). To determine if HmrX is part of the same signaling pathway as HmrA, we constructed a strain harboring an in-frame deletion of *hmrX*, and tested its dispersal through semisolid agar, adhesion, and holdfast production capabilities (Fig. 7B through D).

The ∆*hmrX* mutant displayed a smaller swim diameter in semisolid agar plates compared to WT (Fig. 7B). This phenotype was fully restored to WT levels when the mutant was complemented with *hmrX* (Fig. 7B). In addition, biofilm formation and the number of cells bearing a holdfast were increased in the ∆*hmrX* mutant (Fig. 7C and D). In summary, deletion of *hmrX* yielded phenotypes that were similar to those of the ∆*hmrA* mutant, although their amplitude was less pronounced. Interestingly, the ∆*hmrA* ∆*hmrX* double mutant displayed swimming and biofilm phenotypes that were intermediate between the strong ∆*hmrA* phenotype and the more modest ∆*hmrX* phenotype. Regarding the ratio of cells harboring holdfast, ∆*hmrX* was epistatic to *hmrA*, suggesting that the putative HPT HmrX is part of the Hmr pathway, which is also supported by its identification as a suppressor of ∆*hmrA*.

**Fig 8:**
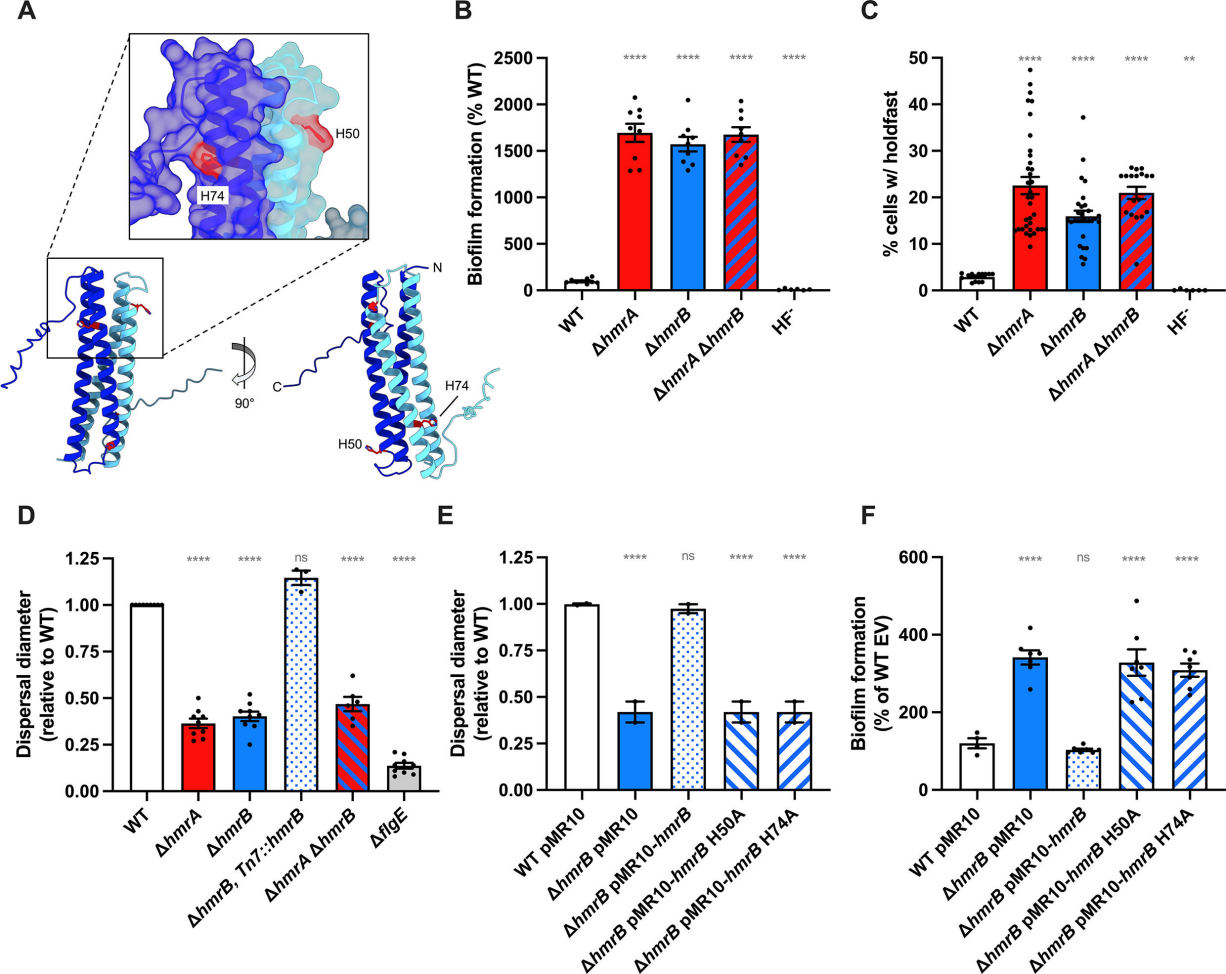
HmrB, a putative atypical HPT involved in the Hmr pathway. (**A**) HmrB dimer structure predicted by AlphaFold-multimer as implemented within ColabFold. The putative catalytic residues H50 and H74 are shown in red. (**B**) Biofilm formation in 24-multiwell plates after 24 h of incubation in M2X medium. Results are expressed as a percentage of biofilm formed by each strain compared to WT set at 100%. Results are given as the average of three independent experiments, each run in triplicate, and the error bars represent the SEM. Statistical comparisons are calculated using one-sample *t* tests to determine if the mean of each sample differs significantly from 100. (**C**) Quantification of cells harboring a holdfast. Holdfasts from mixed populations of each indicated strain grown to mid-exponential phase were labeled using AF488-WGA, and the number of cells harboring a holdfast per field of view was quantified by microscopy. The results are given as the percentage of cells fluorescently labeled (bearing a holdfast) over the entire population and represent the average of at least three independent replicates (more than 300 cells per replicate) with SEM. Statistical comparisons to WT are calculated using ANOVA and a Benjamini-Hochberg protocol for multiple comparisons. (**D and E**) Motility assays through M2X semisolid agar. Swim rings obtained after 5 days of incubation at room temperature were measured and results were normalized to WT ring diameter measured on the same plate (set to 1). Bar graphs indicate the mean of three independent replicates, and error bars represent SEM. Statistical comparisons to WT are calculated using one-sample *t* tests to determine if the mean of each sample differs significantly from 1. (**F**) Biofilm formation in 24-multiwell plates, after 24 h of incubation in M2X medium. Results are expressed as a percentage of biofilm formed by each strain compared to WT set at 100%. Results are given as the average of three independent experiments, each run in triplicate, with SEM. Statistical comparisons to WT are calculated using one-sample *t* tests to determine if the mean of each sample differs significantly from 100. ns: not significant; **P < 0.01; ****P < 0.0001.

Furthermore, although *CCNA_03325* (*hmrB*) was not identified in our genetic screens, we investigated its function since it is adjacent to *hmrA* and *hmrC* (Fig. 1C and 2A) and its protein product meets two of the three criteria previously used to search for HPT proteins in *C. crescentus* (50): it is less than 250 aa (108 aa), and is predicted to contain more than 70% alpha helices (87%). However, the HxxKG motif, used as a third criterium, is not present in HmrB, but it is not clear if this motif is a reliable signature of HPTs (51). The AF2-predicted structure (AFDB A0A0H3CBB1) suggests that HmrB forms an α-helical hairpin with no meaningful similarity to experimentally resolved structures as determined by Foldseek. Given these findings, we wondered whether HmrB may form a more biologically relevant higher-order oligomer, and so utilized AF2-multimer implemented within ColabFold to explore this. This analysis revealed that HmrB most likely adopts a dimeric arrangement that recapitulates the four-helix bundle typically observed in HPT proteins (Fig. 8A; Fig. S8). Formation of a functional HPT domain via dimerization of an α-helical hairpin motif has previously been observed for *Bacillus subtilis* Spo0B (52) and *C. crescentus* ChpT (53), therefore it is possible that HmrB is another example of this type of atypical HPT.

Phenotypic analysis showed that the ∆*hmrB* mutant displays (i) a strong hyperbiofilm phenotype (Fig. 2A and 8B), (ii) a high ratio of cells harboring a holdfast (Fig. 8C), and (iii) impaired swimming motility through semisolid agar (Fig. 8D). These three phenotypes are comparable to those of the ∆*hmrA* mutant and can be reverted to WT levels by complementation with *hmrB* under its native promoter (Fig. 2A and 8D). The ∆*hmrA* ∆*hmrB* double mutant behaved similarly to the ∆*hmrA* and ∆*hmrB* single mutants (Fig. 8B through D), suggesting that HmrA and HmrB act in the same pathway. Finally, assuming that HmrB is an atypical HPT, its phosphotransferase activity could be achieved by one of the two histidine residues, H50 and H74. The predicted three-dimensional structure indicates that both H50 and H74 are solvent accessible (Fig. 8A), thus, both are candidates for the catalytic residue for phosphotransfer. Both histidine residues were substituted with alanine and neither of the substituted versions could rescue the biofilm formation or swimming motility phenotypes of the ∆*hmrB* strain (Fig. 8E and F). In aggregate, these findings suggest that *hmrB* codes for an atypical HPT that acts in concert with HmrA.

### HmrC is required for sensing environmental stress and functions upstream of HmrA

**Fig 9:**
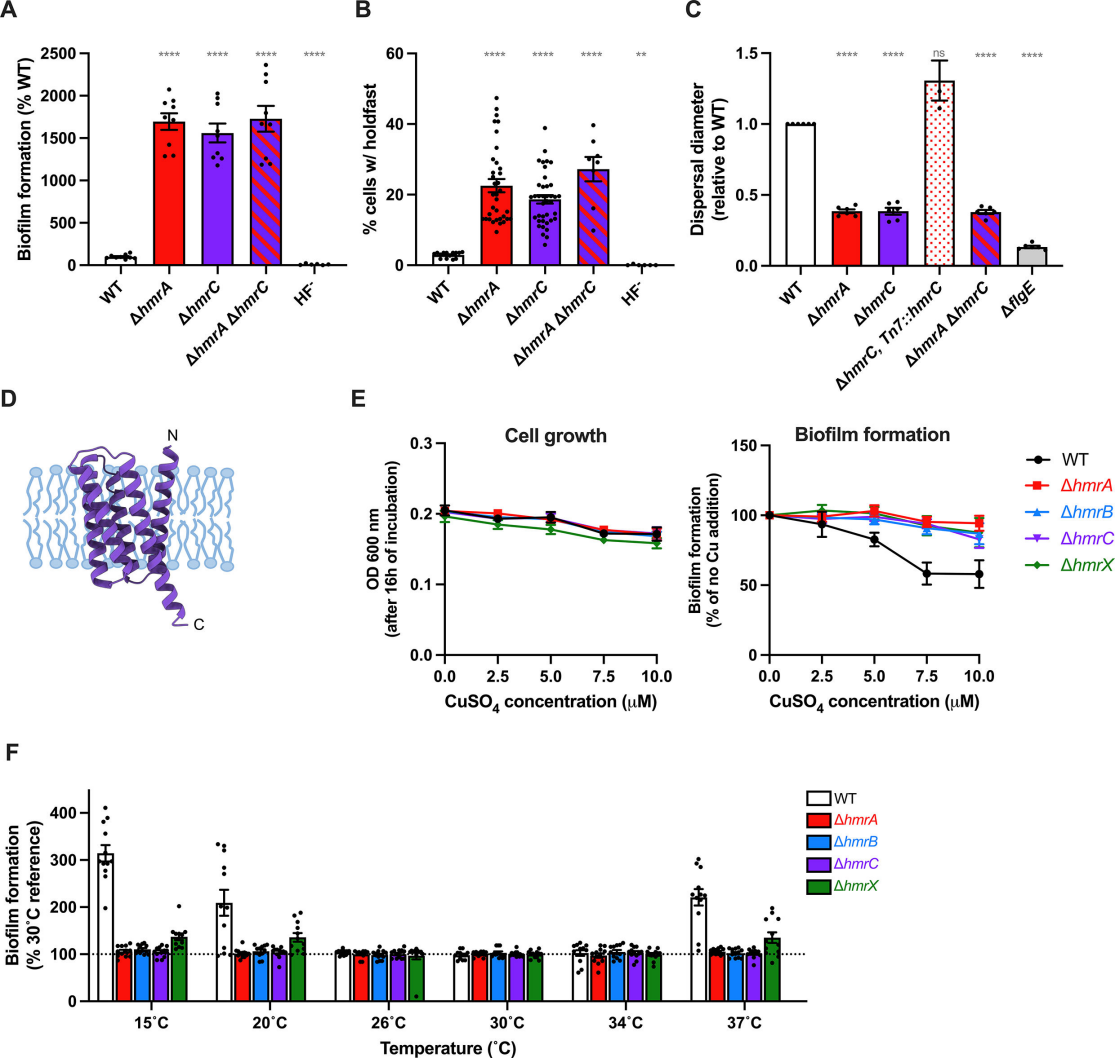
HmrC, a putative membrane protein sensing environmental stress involved in the Hmr pathway. (**A**) Biofilm formation in 24-multiwell plates after 24 h of incubation in M2X medium. Results are expressed as a percentage of biofilm formed by each strain compared to WT set at 100%. Results are given as the average of three independent experiments, each run in triplicate, with SEM. Statistical comparisons are calculated using one-sample *t* tests to determine if the mean of each sample differs significantly from 100. (**B**) Quantification of cells harboring a holdfast. Holdfasts from mixed populations of each indicated strain grown to mid-exponential phase were labeled using AF488-WGA, and the number of cells harboring a holdfast per field of view was quantified by microscopy. The results are given as the percentage of cells fluorescently labeled (bearing a holdfast) over the entire population, and represent the average of at least three independent replicates (more than 300 cells per replicate) with SEM. Statistical comparisons to WT are calculated using ANOVA and a Benjamini-Hochberg protocol for multiple comparisons. (**C**) Motility assays through M2X semisolid agar. Swim rings obtained after 5 days of incubation at room temperature were measured and normalized to WT ring diameter measured on the same plate (set to 1). Bar graphs indicate the mean of three independent replicates with SEM. Statistical comparisons to WT are calculated using one-sample *t* tests to determine if the mean of each sample differs significantly from 1. (**D**) AlphaFold predicted structure of HmrC (AFDB A0A0H3CEK9); with membrane localization predicted by DeepTMHMM (**E**) Overnight growth (left) and biofilm formation (right) for WT (black circles), ∆*hmrA* (red squares), ∆*hmrB* (blue triangles), ∆*hmrC* (purple upside down triangles), and ∆*hmrX* (green diamonds). Samples were grown in 24-multiwell plates after 24 h of incubation in M2X medium, with various concentrations of CuSO_4_. Biofilm results are expressed as a percentage of biofilm formed by each strain in the presence of metal compared to the no metal addition set at 100%. Results are given as the average of eight independent experiments, each run in triplicate, and the error bars represent SEM. (**F**) Biofilm formation in M2X medium using different incubation temperatures. Results are expressed as a percentage of biofilm formed by each strain at a given temperature compared to the 30°C reference set at 100%. Results are given as the average of three independent experiments, each run in triplicate, and the error bars represent SEM. ns: not significant; **<*P* < 0.01; ****P < 0.0001.

Finally, we analyzed *hmrC*, the third gene in the *hmrCBA* cluster and the site of two transposon insertions in our initial genetic screen (Fig. 1C; Table 1). In-frame deletion of this gene yielded increased biofilm formation, a higher ratio of cells harboring a holdfast, and decreased swimming through semisolid agar, all at comparable levels to ∆*hmrA* and ∆*hmrB* (Fig. 2A and 9A through C). Performing these experiments with the ∆*hmrA* ∆*hmrC* double mutant resulted in the same phenotypes as with the single ∆*hmrA* and ∆*hmrC* mutants. From this set of experiments, we propose that HmrA and HmrC function in the same regulatory pathway.

Genome annotation did not ascribe a function to HmrC and a BLAST search did not return any conserved domain. The AF2-predicted structure of HmrC (AFDB A0A0H3CEK9) contains five consecutive α-helices, all of which are predicted to be transmembrane helices by DeepTMHMM (28), with short loops of less than ten amino acids connecting them (Fig. 9D). The I-TASSER server for prediction of protein structure and function (54) revealed that *hmrC* encodes a protein with similarity to the substrate binding component (S-component) of an energy-coupling factor (ECF) type transporter (55). Finally, analysis of the predicted three-dimensional structure from AlphaFold with the Dali server returned hits involved in signal sensing/transducing and phosphotransfer.

Previous whole-genome transcriptome analyses revealed that *C. crescentus hmrC* is regulated by environmental stresses, namely metal exposure and cold shock (56, 57). Indeed, the UzcR response regulator component of the two-component system UzcRS, which is involved in the sensing of U, Zn, and Cu in *C. crescentus*, upregulates *hmrC* by interacting with its promoter (56). Thus, we hypothesized that the Hmr pathway plays a role in growth in the presence of these metals. We tested the ability of WT and all the Hmr mutant strains to make biofilms in the presence of increasing concentrations of copper (CuSO_4_) and zinc (ZnSO_4_). We identified CuSO_4_ and ZnSO_4_ concentration ranges that affect biofilm formation without impacting cell growth for subsequent testing (Fig. 9E; Fig. S9 and S10). While WT biofilm formation is impaired by increased concentration of CuSO_4_ from 5 to 10 µM, it is enhanced by the addition of up to 100 µM ZnSO_4_ (Fig. 9E; Fig. S9 and S10). However, the Hmr mutants all behave similarly and fail to respond to the addition of these metals (Fig. 9E; Fig. S9 and S10). While the differences between WT and the Hmr mutants were statistically significant for CuSO_4_ experiments, it is not the case for the ZnSO_4_ experiments. This could be explained by the variability in the response to metal addition that was observed during these experiments, with the amplitude of the WT response to metals strongly fluctuating between replicates. Thus, we conclude that the Hmr pathway modulates biofilm formation in the presence of excess metals, especially excess copper.

**Fig 10:**
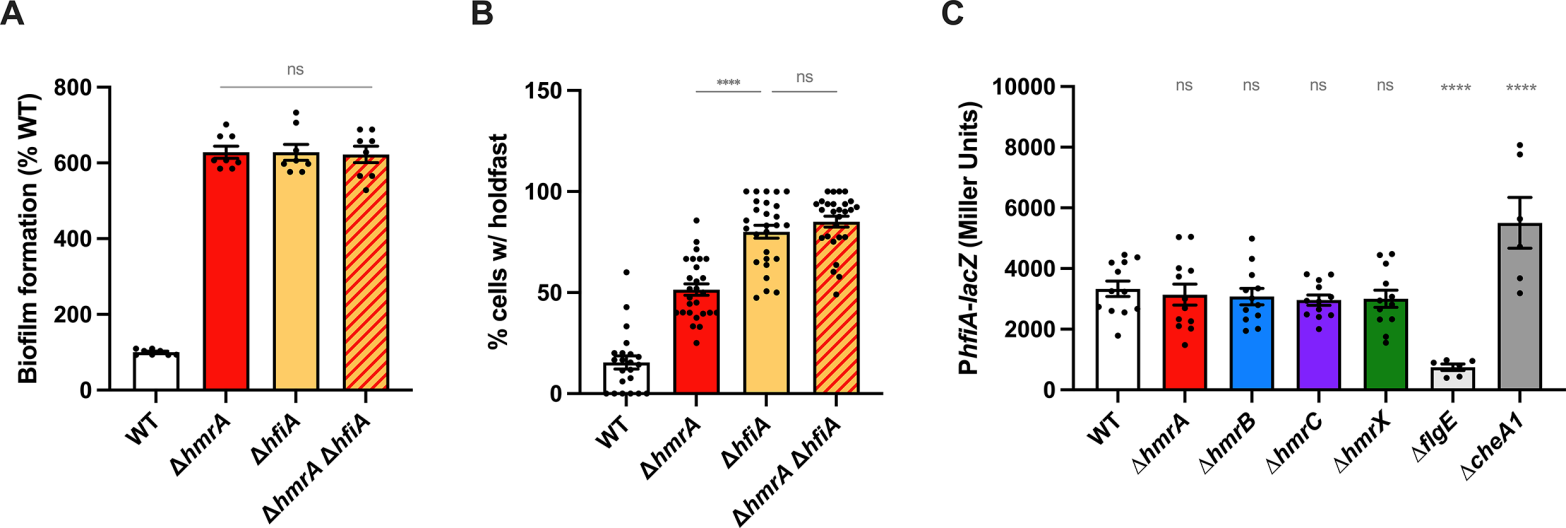
The holdfast inhibitor HfiA is not involved in the HmrA-mediated holdfast regulation pathway. (**A**) Biofilm formation in 24-multiwell plates after 24h of incubation in M2X medium. Results are expressed as a percentage of biofilm formed by each strain compared to WT set at 100%. Results are given as the average of three independent experiments, each run in triplicate with SEM; one-sample *t* tests were used to determine if the mean of each sample differs significantly from 100. (**B**) Quantification of cells harboring a holdfast in mixed populations. Holdfasts from mid-exponential phase cultures were labeled using AF488-WGA, and the number of cells harboring a holdfast per field of view was quantified by microscopy. The results are given as the percentage of cells fluorescently labeled (bearing a holdfast) over the entire population calculated with three independent replicates (more than 250 cells per replicate) and the error bars represent the SEM. (**C**) ß-galactosidase activity of the P*_hfiA_-lacZ* transcriptional fusions in WT and ∆*hmrA* strains. Results are given as the average of three samples (assayed on four different days) and the error bars represent the SEM. For panels **B** and **C**, statistical comparisons are calculated using ANOVA and a Benjamini-Hochberg protocol for multiple comparisons. ns: not significant; **P < 0.01; ****P < 0.0001.

The *hmrC* gene was also found to be upregulated in response to cold exposure by RNA-seq (57). Therefore, we decided to monitor the biofilm formation of WT and the whole set of *hmr* mutants at a range of temperatures, from 15°C to 37°C (Fig. 9F; Fig. S11). We noticed that at temperatures of 15°C, 20°C, and 37°C, WT forms around two to three times more biofilm compared to temperatures closer to the optimal growth temperature of *C. crescentus* (26°C, 30°C, and 34°C) (Fig. 9F; Fig. S11). However, there are minimal differences for the Hmr mutants: biofilm formation is similarly derepressed regardless of the tested temperatures (Fig. 9; Fig. S11). These results suggest that the Hmr pathway plays a role in regulating adhesion as a function of temperature by maximally repressing adhesion at the optimal growth temperature; this repression decreases as the temperature deviates from 30°C. Taken together, we propose that the Hmr pathway regulates adhesion in response to environmental factors, such as the presence of excess copper and growth temperatures, with HmrC acting as the environmental sensor.

### The regulation of holdfast synthesis by the Hmr pathway is independent of *hfiA* transcription

We showed above that the Hmr pathway is involved in regulating holdfast production under some environmental stresses. It has been shown previously that the protein HfiA acts as a key regulator of holdfast synthesis under various stressful environmental conditions such as nutrient limitation or blue light exposure (15). HfiA is regulated at different levels to ensure a proper level of holdfast synthesis and its expression is controlled by a complex network of TCS including several HHKs (15, 20). Therefore, we wanted to determine whether the Hmr pathway could be involved in HfiA regulation.

We first measured biofilm formation using WT, ∆*hmrA*, ∆*hfiA*, and a ∆*hmrA* ∆*hfiA* double-mutant strain. Our results showed a similar increase for all mutants compared to WT (Fig. 10A). To have a closer look at holdfast production, we then quantified the percentage of cells with a holdfast in mixed populations. A threefold increase in the proportion of cells harboring a holdfast was observed in the ∆*hmrA* culture compared to WT (Fig. 10B). In both the ∆*hfiA* and ∆*hmrA* ∆*hfiA* populations, this number increased fivefold, indicating that HfiA still represses biofilm formation in a ∆*hmrA* mutant. In addition, we measured the expression of *hfiA* using *lacZ* transcriptional fusions to the *hfiA* promoter in WT and all the Hmr pathway mutants. As controls, we also measured the expression of *hfiA* in a ∆*flgE* and a ∆*cheAI* strain, as changes in *hfiA* expression have been previously reported in these strains [downregulation in the ∆*flgE* mutant (38) and upregulation in the ∆*cheAI* mutant (16)]. Fig. 10C shows that the activity of the *hfiA* promoter P*_hfiA_* is similar in the WT, ∆*hmrA*, ∆*hmrB,* ∆*hmrC*, and ∆*hmrX* strains. These results indicate that the Hmr proteins do not regulate *hfiA* transcription.

### Conclusion

**Fig 11:**
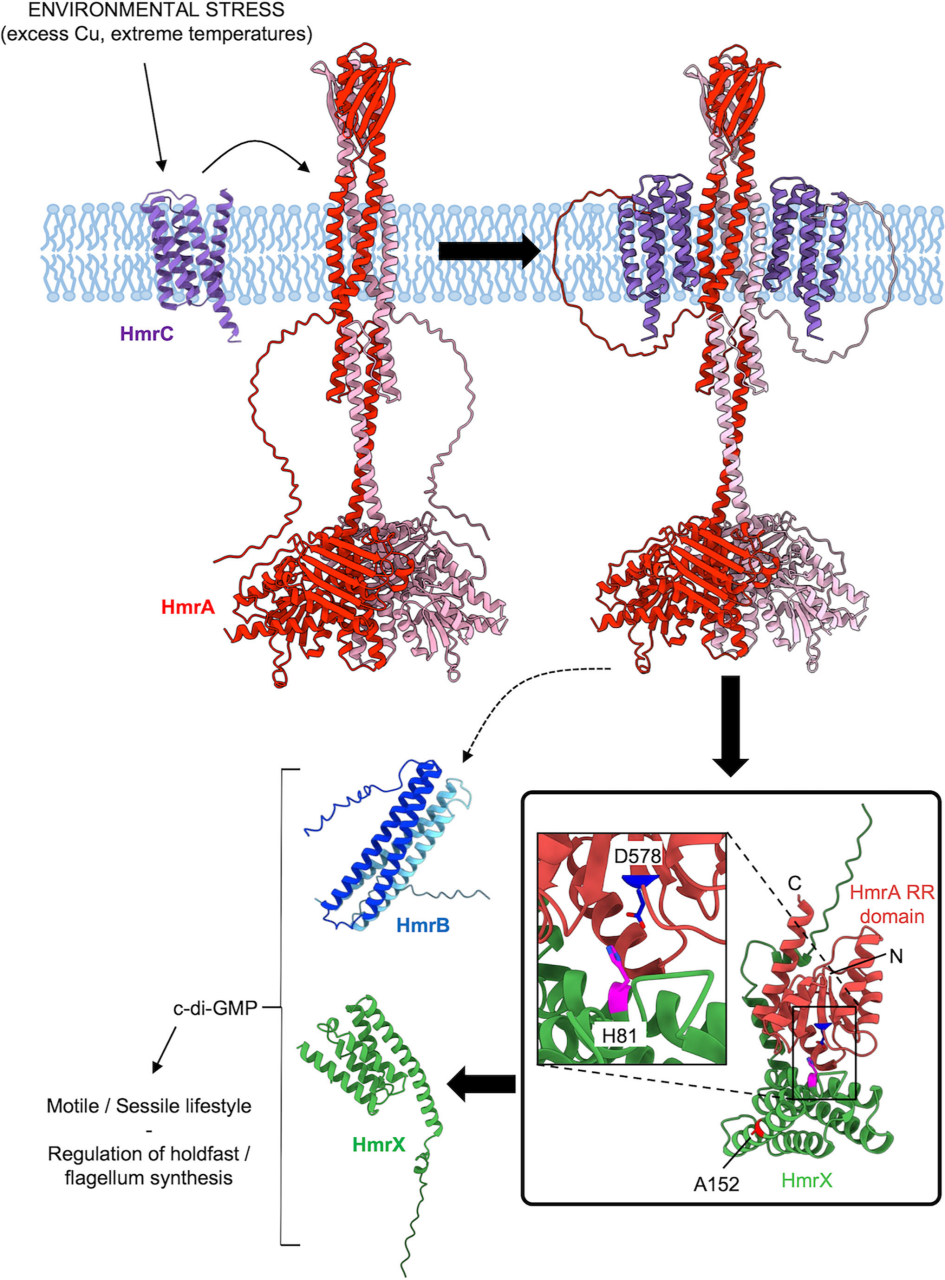
Proposed model for the Hmr pathway. Presence of an environmental stress (such as excess copper or extreme temperature) is detected by the membrane protein HmrC, which transfers the signal to the HHK HmrA. This signal is transduced to the HPT proteins HmrB and HmrX, which results in a change in c-di-GMP levels via the action of DgcB and PdeA. Changes in c-di-GMP levels modify the regulation of flagellum and holdfast synthesis and dictate the switch from the motile to sessile state for each individual cell.

From the above results, we postulate that the *hmrABC* gene cluster, along with *hmrX*, encodes different elements of a phosphorelay involved in the regulation of motile versus sessile lifestyles independently of *hfiA* transcription. This phosphorelay consists of a transmembrane HHK HmrA, two HPTs (HmrB and HmrX), and a membrane protein (HmrC) at the top of the signaling hierarchy, sensing environmental stress (Fig. 11). In this model, excess copper or extreme temperatures are sensed by HmrC, which transmits a signal to HmrA and triggers changes in c-di-GMP levels via HmrX and HmrB. This results in a change in the number of cells bearing a flagellum or a holdfast in the population. We propose that the Hmr pathway detects environmental stresses and acts as a rheostat by promoting cells to disperse (more cells with a flagellum) while discouraging adhesion (less cells with a holdfast). This regulation allows the population to respond to its environment by adjusting the settling/dispersing ratio accordingly.

## DISCUSSION

HmrA (CC3219/CCNA_03326) was first reported in a genome-wide study to identify HHKs in *C. crescentus* (58), which reported that a ∆*CC3219* mutant is impaired in swimming through semisolid agar. We show here that, in addition to impacting group swimming, HmrA is also important for adhesion and biofilm formation, by fine-tuning the number of cells producing a holdfast in the population. Future biochemical analysis will be required to confirm that the actors of this putative phosphorelay can indeed directly phosphorylate each other, and in which sequence. We showed that residues H286 and D578 in HmrA are crucial for both inhibiting biofilm formation and promoting swimming dispersal. We could not evaluate whether the loss of activity resulted specifically from amino acid substitutions that eliminated catalytic activity, or from an overall structural destabilization. However, since these mutations have been analyzed multiple times in HK research and typically do not destabilize HK structure, we interpret these phenotypes as good indications that *hmrA* encodes a functional HHK. In addition, the most downstream element of the phosphorelay is currently unknown and its identification will be required to complete our knowledge of this pathway.

It is worth noting that the initial screen that enabled the identification of the Hmr pathway revealed two HHKs: HmrA and CCNA_03265 (CC3162) (Fig. 1; Table 1). Interestingly, CCNA_03265 was also shown to impact group motility through semisolid agar (58). Sequence alignment and structure analysis indicate that the CCNA_03265 HK domain lacks the conserved histidine while the RR domain has retained the aspartate residue (Fig. S3). One possibility would be that the catalytic histidine does not perfectly align with the sequences of other HHKs but is still present in the vicinity. Located a few aa downstream on the same alpha helix, H151 is a reasonable candidate to fulfill this function (Fig. S3 and S12). Another possibility would be that CCNA_03265 has no catalytic histidine and that its RR aspartate is phosphorylated by an HPT, such as HmrB or HmrX, or forms a heterodimer with another HHK, such as HmrA. The model of the canonical isolated, linear phosphorelay has prevailed for a long time, but more recent work has shown that phosphorelays can be more complex. For example, in *C. crescentus*, several kinases can phosphorylate the single domain response regulator MrrA (17), which in turn can phosphorylate both the PhyK/PhyR and the LovK/LovR systems known to be involved in general stress response (59), cell cycle regulation via HfiA (15), and holdfast production (14). In *P. aeruginosa*, the GacS-GacA TCS (60) interacts with three other HHKs, RetS, LadS, and PA1611, to control the transition between motile and sessile lifestyles depending on the environment (61–63). These examples of regulation cascades involving multiple inputs illustrate the complex molecular mechanisms underlying how bacteria adapt to the different environmental conditions they encounter and how they regulate their lifestyle in response to these changes. Future work could help to decipher if CCNA_03265 (i) is in the same phosphorelay as HmrA; (ii) can interact with HmrA; and (iii) regulates the transition between motile to sessile lifestyles upon different signals.

### The Hmr pathway is important for tuning biofilm formation in response to environmental stresses

Our results suggest that the Hmr pathway is important for adapting the motile to sessile transition in response to environmental cues, as HmrA, HmrB, HmrC, and HmrX are involved in the response to environmental stresses such as the presence of excess metals or non-optimal temperatures (Fig. 9E and F; Fig. S9 through S11). *hmrC* was shown to be upregulated in the presence of metals and is part of the UzcR regulon, which is involved in U, Zn, and Cu sensing (56). We tested various metals, namely Cu, Zn, Co, Fe, Mn, and Ni. In the tested conditions, only Cu excess produced a significant alteration of the biofilm phenotype in WT cells. While the effect of Cu on biofilm formation is significant and the Hmr pathway is involved, we cannot rule out that this regulation is indirect and results from a general overproduction of biofilm. A recent RNAseq study showed that *C. crescentus* reacts to Cu excess by overexpressing machineries dealing with Cu detoxification (PcoAB), oxidative stress, protein misfolding/rearrangement, and chelation by cysteine and arginine (64). In the same study, it stands out that *hmrC* is the only gene of the *hmr* set that is consistently upregulated by the addition of Cu (2.4× in stalked cells; 4.1× in swarmer cells). Other *hmr* genes displayed either no change or a twofold downregulation upon Cu addition. Considering our results, HmrC is an important factor in managing the response to excess copper. We show here that biofilm formation in *C. crescentus* is decreased by the addition of Cu, while it remains steady in the Hmr mutants, suggesting that this pathway is important to modulate biofilm adhesion under Cu stress. The cell type of *C. crescentus* (swarmer versus stalked cells, Fig. 1A) determines the response to Cu excess: while swarmer cells preferentially escape excess copper by negative chemotaxis, stalked cells are seemingly more impacted by Cu stress and rely on detoxification mechanisms (primarily PcoAB) (65). The different fate for each cell type was postulated to condition a fast bimodal response to Cu stress (65). Since HmrC, and the Hmr pathway in general, appears to determine the proportion of motile versus sessile cells in a mixed population, we hypothesize that the Hmr pathway, with its response to Cu levels, might be a molecular mechanism underlying this bimodal response by controlling the balance between motile (swarmer cells bearing a flagellum) and sessile (stalked cells lacking a flagellum) lifestyles at the population level.

*C. crescentus* has an optimal growth temperature of 30°C. Our results show that it produces approximately two to three times more biofilm at 15°C, 20°C, and 37°C. This difference depends on all of the Hmr proteins, which repress biofilm formation as a function of temperature with a maximum repression at 30°C and milder repression for non-optimal temperatures (Fig. 9F). Interestingly, *hmrC* is upregulated during cold shock, which affects metal and ion homeostasis (57). It is not clear at this stage whether HmrC is involved in sensing broad environmental stress, including metal-induced *and* cold-induced stress, or whether it is involved in sensing environmental metals and its role in cold sensing is a consequence of the disturbance of metal homeostasis. Temperature changes can destabilize the bacterial membrane by changing its fluidity. In the case of cold shock, the main signal that triggers adaptation is the sensing of membrane rigidity by transmembrane histidine kinases, as described in a broad range of organisms such as *E. coli*, *B. subtilis*, *S. typhimurium*, *Yersinia pestis,* and *Synechocystis* PCC6803 (66). Based on our results, we hypothesize that the membrane protein HmrC is required for sensing temperature changes, which conditions the proportion of biofilm-forming cells as a function of the temperature. Interestingly, deletion of *hmrC* comes at a high fitness cost according to a genome-wide gene essentiality study in *C. crescentus* (67). Overall, this could point to HmrC as a crucial element for sensing environmental stresses. Since HmrC and HmrA both reside in the inner membrane and likely function in the same signaling pathway, HmrC may interact with HmrA to facilitate this process, as has been observed for other HK’s with membrane integral accessory proteins (68).

Based on the above considerations, we utilized AF2-multimer as implemented in ColabFold to predict whether a complex between a HmrA dimer and HmrC would be feasible. This generated a set of consistent and confidently predicted models wherein two copies of HmrC were positioned on either side of the HmrA dimer transmembrane region, interacting with one of the two HmrA chains in the dimer (Fig. 11 ; Fig. S13). Since relatively small changes in the conformation of transmembrane helices can be amplified via adjacent HAMP domains to downstream cytosolic modules (34), this may represent a mechanism whereby signals linked to HmrC sensing are transmitted into the cell via HmrA (Fig. 11). We can then speculate that HmrA participates in a phosphorelay with HmrB and/or HmrX as intermediaries. Indeed, the alignment of the AF2-predicted structure of HmrX with the P1 domain of *E. coli* CheA superimposes the substrate histidine of CheA with H81 of HmrX, pointing to a potential site of phosphorylation. Modeling of HmrX with the response regulator domain of HmrA using AF2-multimer (Fig. 11; Fig. S14) positions the predicted catalytic aspartate of HmrA in close proximity to H81 of HmrX (Fig. 11) in an arrangement reminiscent of other response regulator-HPT structures (69, 70), supporting the hypothesis that HmrA and HmrX participate in a phosphorelay. HmrX and/or HmrB then eventually modulate the transition from motile to sessile lifestyles and ensure that cells settle in favorable environments. Finally, the phenotypes of ∆*hmrX* suggest that other pieces of the relay remain to be discovered. Indeed, the less pronounced phenotype of ∆*hmrX*, compared to the mutants related to its presumed upstream partners (∆*hmrC* and ∆*hmrA*), indicates that an unknown phosphotransfer may occur in parallel to that mediated by HmrX. Furthermore, the intermediate intensity of the ∆*hmrA* ∆*hmrX* phenotypes, compared to those of ∆*hmrA* and ∆*hmrX*, shows that disrupting *hmrX* mitigates the strong phenotype modification induced by the deletion of *hmrA*. At this stage, it is not possible to explain these phenotypes, but they clearly reflect the yet to be discovered complexity of the Hmr pathway, which is likely to branch with other pathways.

### c-di-GMP, produced by DgcB, is involved in the Hmr pathway

We show in this work that the Hmr regulatory pathway likely impacts the level of intracellular c-di-GMP, via the diguanylate cyclase DgcB to control both holdfast and flagellum production. Indeed, two different suppressor screens of ∆*hmrA* for the gain of swimming through semisolid agar (Fig. 5) identified multiple independent transposon insertions and point mutations in *dgcB*, suggesting that the Hmr pathway and the level of c-di-GMP are linked. This model is in line with the well-established role of c-di-GMP as an important player in the motile to sessile lifestyle transition in *C. crescentus* (71). In addition, our transposon screen for hyper-adhesive mutants (Fig. 1B) enriched for five different mutants in the phosphodiesterase encoding gene *pdeA* (Fig. 1C; Table 1). PdeA affects motility and biofilm formation through c-di-GMP and the activity of DgcB is counteracted by PdeA (13). PdeA is degraded during the motile swarmer to sessile stalked cell transition (72) and DgcB is then able to contribute to increasing c-di-GMP levels in transitioning cells. Stimulation of DgcB activity is achieved by several players at the motile to sessile turning point. First, when swarmer cells encounter a surface in complex medium, pili and the flagellum motor are used as mechanosensors to trigger DgcB to synthesize c-di-GMP, which promotes holdfast synthesis (19, 37). In addition, the CheY-like c-di-GMP effectors proteins CleA and CleD are activated by c-di-GMP and interact with the flagellar motor to promote surface contact-stimulated production of holdfast (44). Finally, the flagellar stator MotB and the flagellar signaling suppressor proteins FssA and FssB also trigger DgcB-related c-di-GMP production via the mechanical pathway of holdfast synthesis, regardless of the presence of a surface, via modulation of HfiA expression (48). In those cases, the production of c-di-GMP via DgcB is believed to lead to the activation of the holdfast synthesis protein HfsJ, which binds to c-di-GMP. Our work on the Hmr pathway provides insights into a different regulation mechanism because: (i) we performed our experiments in M2X minimal medium where surface contact stimulation of holdfast synthesis is not active (38); and (ii) we showed that HfiA is not involved in the Hmr pathway. Future work could provide more insight into whether HfsJ is the final player in the Hmr regulation cascade.

### The Hmr pathway regulates motile to sessile lifestyle transition independently of *hfiA* trancription

Several TCS have been shown to regulate holdfast production in *C. crescentus*. For example, the general stress-response PhyR/NepR (17), the LOV (light, oxygen, and voltage) blue light photoreceptor proteins LovK/LovR (59), and the RegB/RegA homologs SpdS/SpdR (73) all control holdfast synthesis. They act by regulating the transcription of *hfiA*, the major regulator of holdfast synthesis, itself regulated via a multilayered network (15, 20). Interestingly, our work shows that the Hmr pathway regulates holdfast production without modulating *hfiA* transcription (Fig. 10), revealing a novel facet of the highly complex network dedicated to holdfast regulation. However, we cannot rule out potential post-transcriptional regulation of HfiA by the Hmr pathway, as is the case with the chaperone DnaK (18).

A previous ChiP-Seq analysis reported that the cell cycle regulator GcrA, which was shown to bind to the *hfiA* promoter (15), can also interact with the *hmrB* promoter (74). Thus, the connection between GcrA and the Hmr pathway appears as another interesting future direction to investigate how this cell cycle regulator impacts holdfast production via both the Hmr and HfiA pathways.

## MATERIALS AND METHODS

### Bacterial strains and growth conditions

All bacterial strains used in this study are listed in Table S1. *E. coli* strains used for cloning were grown in LB medium at 37°C in the presence, when necessary, of antibiotics at the following final concentrations: kanamycin 50 µg mL^−1^ (for pMR10 constructs), gentamicin 20 µg mL^−1^ (for pTNS3 constructs), and tetracycline 15 µg mL^−1^ (for pRKlac290 constructs). *C. crescentus* strains were grown at 30°C using M2 minimal medium with 0.2% xylose (M2X) (75) in liquid culture, and using Peptone Yeast Extract (PYE) (76) +15 g L^−1^ bactoagar (Difco) plates. When necessary, antibiotics were added to *C. crescentus* cultures at the following final concentrations: kanamycin 20 µg mL^−1^ (for pMR10 constructs), gentamicin 50 µg mL^−1^ (for pTNS3 constructs), and tetracycline 2 µg mL^−1^ (for pRKlac290 constructs).

### Plasmid construction and cloning procedures

All plasmids were cloned using standard molecular biology techniques. PCR was performed using *C. crescentus* NA1000 *hfsA*^+^ WT genomic DNA as the template. Sequences of the primers used are available upon request.

In-frame deletion mutants were obtained by double homologous recombination, as previously described (77). Briefly, 500 bp fragments from the upstream and downstream regions of the gene to be deleted were amplified by PCR. PCR fragments were gel-purified and then digested by *Hind*III and *BamH*I or *BamH*I and *EcoR*I for upstream or downstream fragments, respectively. Purified digested fragments were then cloned into the suicide vector pNPTS139 that had been digested by *EcoR*I and *Hind*III. The pNPTS139-based constructs were transformed into *E. coli* DH5a cells and then introduced into *C. crescentus* by electroporation. The two-step recombination was carried out using sucrose resistance and kanamycin sensitivity (77). Then, the mutants were verified by sequencing to confirm the deletion.

The pMR10-*hmrA* and pMR10-*hmrB* constructs were created as follows. The genes and their putative native promoter (approximately 500 bp upstream of the start codon) were amplified by PCR, gel-purified and then digested by *EcoR*I and *BamH*I. Purified digested fragments were ligated into pMR10 that had been digested with *EcoR*I and *BamH*I. Ligation products were used to transform *E. coli* DH5a cells by heat shock. Once plasmids were isolated from *E. coli* and the sequence of the insert was confirmed through Sanger sequencing, plasmids were electroporated into *C. crescentus. C. crescentus* cells harboring the pMR10 derivatives were screened for kanamycin resistance. The point-mutation derivatives of these strains were generated using oligos containing the appropriate point mutation.

The complementation of mutants in *hmrA, hmrB, hmrC*, and *hmrX* was performed by inserting each gene with its promoter at the Tn7 *att* site. Briefly, a region consisting of the gene of interest was amplified with flanking regions to preserve the promoter and terminator sequences (approximately 300 bp upstream and 100–200 bp downstream). The PCR product was subsequently inserted into pUC18-mini-Tn7-LAC (78). The resulting plasmid was introduced in the *C. crescentus* strain of interest along with the helper plasmid pTNS3 (79) to insert the gene of interest under its native promoter at the Tn7 *att* site in the chromosome of *C. crescentus*. This insertion contains a cassette that confers resistance to gentamicin, which enables selection.

*C. crescentus* NA1000 *hfsA^+^* derivatives harboring the stable miniTn7*dsred* were constructed by ΦCR30 mediated transduction (80) from CB15::miniTn7*dsred* (YB4788) lysate as described previously (81). The NA1000 *hfsA^+^ divJ-cfp, pleC-yfp* strains were constructed by ΦCR30 mediated transduction (80) from CB15N *divJ-cfp, pleC-yfp* lysate where the chromosomal copies of *divJ* and *pleC* were replaced with *divJ-cfp* and *pleC-yfp* (82).

### Motility assays through semisolid medium

Motility assays were performed using semisolid agar plates. Plates were poured using M2 medium supplemented with 0.2% (wt/vol) xylose and 0.4% (wt/vol) noble agar (Difco) and allowed to sit overnight at room temperature. Cells from colonies grown on regular PYE agar were stabbed into the soft agar and incubated in a humid chamber at 30°C for 3 days or room temperature for 5 days. The diameter of the swimming ring formed by each tested strain was measured manually and normalized to that of WT (with empty vector when appropriate).

### Biofilm assays

Biofilm assays in 24-multiwell plates were performed as described previously (81). Bacteria were grown to mid-log phase (OD_600_ of 0.3–0.6) in M2X and diluted to an OD_600_ of 0.05 in the same medium. A volume of 500 µL of freshly diluted cells were placed in each well. Plates were incubated at 30°C for 24 h, or otherwise as mentioned in the text. Growth inside the 24-well plates (OD_600_) was recorded directly from the wells using a Spectramax ID3 (Molecular Devices) microplate reader. Biofilms attached to the inside surface of the wells were quantified as follows: wells were rinsed with distilled H_2_O to remove non-attached bacteria, stained using 0.1% (wt/vol) crystal violet (CV), and rinsed again with dH_2_O. The CV from the stained attached biomass was eluted using 10% (vol/vol) acetic acid and was quantified by measuring absorbance at 600 nm (*A*_600_). Biofilm formation was normalized to *A*_600_/OD_600_.

### Holdfast quantification using fluorescently labeled WGA lectin

The number of cells harboring a holdfast in mixed populations was quantified by fluorescence microscopy. Holdfasts were detected with AlexaFluor 488 conjugated wheat germ agglutinin (AF488-WGA) since WGA binds specifically to the N-acetylglucosamine residues present in the holdfast (83). Early exponential-phase cultures (OD_600_ of 0.2–0.4) were mixed with AF488-WGA (0.5 µg mL^−1^ final concentration in water). A volume of 1 µL of WGA-stained cells was spotted on a 24 × 60 mm^2^ glass coverslip and covered with an agarose pad (1% [wt/vol] SeaKem LE Agarose dissolved in dH_2_O). Holdfasts were imaged by epifluorescence microscopy. The number of individual cells with a holdfast was calculated manually from microscopy images.

### Holdfast synthesis timing by time-lapse microscopy

Cell division and holdfast synthesis timing were observed in live cells on agarose pads by time-lapse microscopy as described previously (38). A 1 µL aliquot of exponential-phase cells (OD_600_ of 0.4–0.7) was placed on top of a pad containing 1% (wt/vol) SeaKem LE Agarose diluted in M2X and AF488-WGA (0.5 µg/mL final concentration) sealed under a coverslip with valap. Time-lapse microscopy images were taken every 2 min for 8 h. Time-lapse movies were visualized in ImageJ (84) to manually assess the time of cell division, as *t* = 0 when a cell newly divides, the time of holdfast production as the first frame where the fluorescent lectin signal is detected, and the time of new division.

### Imaging and labeling of flagellum filaments

Strains used to visualize flagella by fluorescence microscopy harbored a cysteine knock-in in the major flagellin *fljK* (38), as well as a miniTn7*dsred* insertion, for cell body visualization (81). Cell cultures were grown to mid-exponential phase (OD_600_ = 0.3–0.4) in M2X medium. A volume of 100 µL of culture was mixed with 10 µL of AF488 conjugated maleimide (AF488-mal, 50 µg mL^−1^ in water) for a final dye concentration of 5 µg mL^−1^ and incubated for 5 min at room temperature. Cells were centrifuged at 5,000 × *g* for 1 min, washed with 1 mL of sterile water to remove excess dye and shed flagella, and resuspended in 50 µL of sterile water. A volume of 1 µL of the labeled culture was added to a coverslip and imaged under an agarose pad [1% (wt/vol) SeaKem LE Agarose diluted in water]. Cell bodies and flagella were imaged using red and green fluorescence microscopy settings. The number of cells bearing a flagellum in the mixed population was quantified for microscopy images using ImageJ (84). A flagellated cell was scored as a predivisional cell when its length was greater than 1.5 µm, shorter flagellated cells were scored as swarmer cells.

### Epifluorescence microscopy

Epifluorescence microscopy was performed using an inverted Nikon Ti2 microscope with a Plan Apo 60× objective, a GFP/DsRed filter cube, an Andor iXon3 DU885 EM CCD camera, and Nikon NIS Elements imaging software. Image analysis was performed using the Nikon NIS Elements or ImageJ (84) analysis software built-in tools.

### Nanoscale AFM imaging

A total of 100 µL of cell culture grown to mid-exponential phase (OD_600_ = 0.3–0.4) in M2X medium was diluted in 900 µL of sterile water. A volume of 10 µL was spotted onto a clean glass coverslip and allowed to dry at room temperature. AFM imaging was carried out in air at room temperature using a Bioscope resolve AFM (Bruker) in the peak-force tapping mode, silicon cantilevers with a nominal spring constant of around 0.4 N m^−1^, and a nominal tip radius of 2 nm (ScanAsyst-Air, Bruker).

### Cell synchronization, initial attachment of *C. crescentus* single cells to surfaces, and motile cell swimming speed quantification

Small-scale synchronization of *C. crescentus* cells was performed as described previously (85) with some modifications. Overnight cultures in M2X were diluted 10-fold in 30 mL of fresh M2X and incubated, with shaking at 50 rpm, at room temperature in a 150 mm diameter petri dish. After overnight incubation, the medium was removed and the monolayer biofilm formed in the dish was thoroughly washed with sterile water. A volume of 30 mL of fresh M2X medium was added to the dish and the incubation was carried out for 4 h under the same conditions. The dish was then rinsed 10 times using 10 mL of fresh M2X medium to remove unattached cells from the biofilm. A volume of 1 mL of M2X medium was added to the dish and the incubation was carried out for 5 min under the same conditions to release the newly born swarmer cells. The homogeneity of the synchronized swarmer population was verified by microscopy.

The initial attachment of *C. crescentus* cells to surfaces was recorded by dark-field microscopy as described previously (81). A volume of 1 µL of synchronized swarmer cells were observed by dark-field microscopy (10× objective). A stack of 100 frames (100 ms exposure time) was recorded using RAM capture every minute for 1 h. The maximum intensity of the 20 first frames of each stack was calculated to visualize motile cells with a swimming trajectory, using the built-in functions in ImageJ. The number of swimming trajectories in the field of view was quantified manually and the value calculated for each sample at *t* = 0 was set as 100%.

Recorded RAM capture stacks were also used to determine the trajectories and velocities of swimming cells. The entire tracks (10 s movies) were analyzed using the MicrobeJ plugin (86) in ImageJ. For each strain, more than 500 cell tracks were analyzed.

### Chemotaxis assays

Chemotaxis assays were performed as previously described (45). Plates were poured using M2 (no carbon added) + 0.2% noble agar. A sterile 500 µm diameter filter paper disk containing 50 µL xylose (from a 20% [wt/vol] solution) was placed in the center of the plate, and cells were spotted 1 cm away from the chemoattractant-filled disk. Plates were incubated at 30°C for 48 h. Chemotaxis rings were imaged using a ChemiDoc imaging system (Bio-Rad), and the relative pixel intensity profile through the center of the chemotaxis ring was calculated for each strain.

### *Mariner* transposon mutagenesis

For *Mariner* transposon mutagenesis, the pFD1 plasmid bearing the *Himar1*-based minitransposon (23) was mated into NA1000 *hfsA*^+^ WT or ∆*hmrA*. Mutants bearing the transposon were selected on PYE + kanamycin (20 µg mL^−1^) and screened for hyper-adhesion or improved motility through semisolid agar as described below.

Genomic DNA from mutants of interest was isolated and subsequently used as a template for inverse PCR to identify the insertion site of the *Mariner* transposon. One microgram of genomic DNA was digested with 5 units of Sau3AI. The digested DNA was then self-ligated with T4 DNA ligase. Between 25 and 150 ng of the self-ligated genomic DNA was mixed with the primers (ACGGTATCGATAAGCTTGATATCGA and CAGAGTTGTTTCTGAAACATGGCA) and PCR amplified. PCR fragments were then gel purified and sequenced using amplification from the transposon primer at the Institute for Molecular and Cellular Biology, Indiana University, Bloomington, USA.

### Screen for hyper adhesive mutants

Wild-type *C. crescentus* NA1000 *hfsA^+^* cells were randomly mutagenized with the *Mariner* transposon as described above. Over 15,000 colonies were selected on PYE + kanamycin plates (20 µg mL^−1^), providing greater than 95% genome coverage (87, 88). Colonies were pooled in sets of approximately 150 in 3 mL M2X medium and incubated overnight in plastic 12-multiwell plates with an 18 × 18 mm^2^ glass coverslip added vertically in each well. After incubation, the cells that were bound to the coverslips were used as inoculation for a new culture: coverslips were washed carefully with fresh M2X medium to remove any loosely attached cells, added to a new well containing 3 mL of fresh M2X and incubated for 3 h. After incubation, new sterile glass coverslips were added to the new culture in the wells, and the process was repeated. After two enrichments the cultures were streaked for single colonies. Approximately 300 colonies were randomly picked and subjected to a secondary screen to identify mutants with more than 200% biofilm formation compared to the WT control.

### Screen for ∆*hmrA* suppressor mutations on semisolid agar plates

Two different approaches were taken to isolate suppressor mutations that would restore the motility defect of the ∆*hmrA* mutant in semisolid agar:

- The NA1000 *hfsA^+^* ∆*hmrA* mutant was mutagenized with *Mariner* transposons as described above. Approximately 26,000 colonies were selected on PYE + kanamycin plates (20 µg mL^−1^). This sample size provided greater than 97% genome coverage (87, 88). The colonies were pooled together into a set of 15 pools and normalized to OD_600_ = 0.3. A volume of 2 µL from each of the normalized pools were stabbed into a PYE plate containing 0.3% agar, and incubated at 30°C. After 48 h, cells from the edge of spreading flares were streaked to obtain single colonies. Only one single flare was selected from each initial semisolid plate to avoid isolating siblings, yielding 15 independent suppressors. A fresh inoculation of each isolated suppressor strain in semisolid agar confirmed their phenotype (swimming ring greater than ∆*hmrA* sized rings). To validate that the suppression phenotype was due to the *Mariner* transposon insertion, ΦCR30 lysates of the 15 mutants were created for transduction as described below. These lysates were used to transduce the *Mariner* transposon insertion into the ∆*hmrA* mutant background. An isolated colony from each of the 15 transductants was inoculated into semisolid agar plates. Fourteen out of 15 transduced mutants retained the increased swimming diameter and were studied further. Genomic DNA was extracted and inverse PCR was performed to identify the transposon insertion site in each mutant.
- Random spontaneous motile suppressors were isolated as follows. Eight separate cultures of NA1000 *hfsA^+^* ∆*hmrA* were grown in 5 mL PYE overnight and streaked on PYE plates. One isolated colony from each plate was stabbed into a semisolid PYE plate and incubated at room temperature. After 96 h, cells were isolated from the edge of different flares and streaked on fresh PYE plates. The swimming phenotype of the isolated motility suppressors was confirmed on semisolid PYE plates and only the strains exhibiting a swimming ring greater than that of ∆*hmrA* were carried further for identification. For each suppressor mutant, the *dgcB* gene was first sequenced, and only strains with a WT copy of *dgcB* were sent for Illumina sequencing (3 out of 11 mutants).

### DNA transduction by ΦCR30 phage lysate

Phage lysates were prepared as follows (80). Donor strains of *C. crescentus* were grown overnight at 30°C in PYE. A volume of 100 µL of a 10^−3^ dilution of live ΦCR30 phage and 200 µL of the overnight donor culture were added to molten PYE top agar (0.5% agar), mixed and poured onto the surface of a pre-heated PYE plate. After overnight incubation at 30°C, 5 mL of fresh PYE were added onto the surface of the plate and incubated overnight at 4°C. The remaining PYE liquid was removed from the plate and transferred to an empty 100 mm petri dish and irradiated under a germicidal UV lamp for 5 min. After irradiation, 200 µL of chloroform were added to samples, transferred to a glass screw cap tube and stored at 4°C, protected from light.

Transductions were done by mixing 500 µL of overnight *C. crescentus* recipient culture, 50 µL of UV-irradiated phage lysate (prepared as described above), and 475 µL of fresh PYE. The mixture was incubated for 4 h at 30°C and plated on a PYE + kanamycin plate, to select for transductants harboring a DNA fragment including the *Mariner* transposon.

### ΦCbK phage sensitivity assay for presence of pili

Overnight cultures to be tested were normalized to OD_600_ = 0.6 and serially diluted in steps of 100× to a final concentration of 10^−8^. A volume of 100 µL of ΦCbK phage solution was uniformly absorbed onto the surface of a PYE plate, and 10 µL of culture dilutions were spotted on the plate and allowed to dry prior to incubation at 30°C. The number of colonies was recorded after 2 days of incubation.

### Quantitative PCR to evaluate *Cori/ter* ratios

The relative chromosomal presence of the *Cori* and *ter* regions was determined as described previously (89) with a few modifications. Briefly, the following primer set was used: Cori_fwd (5′-CGCGGAACGACCCACAAACT-3′) and Cori_rev (5′-CAGCCGACCGACCAGAGCA-3′) to amplify a region close to the origin (*Cori*); Ter_fwd (5′-CCGTACGCGACAGGGTGAAATAG-3′) and Ter_rev (5′-GACGCGGCGGGCAACAT-3′), to amplify a region close to the terminus (*ter*). Cells were grown in M2X medium and harvested in the exponential phase. Genomic DNA was extracted using the Monarch genomic DNA extraction and purification kit (New England Biolabs Inc.). The region of *Cori* and *ter* was quantitatively amplified using the Sybr green-based Luna Universal One-step RT-qPCR kit (New England Biolabs Inc.), without the reverse transcriptase enzyme, in a QuantStudio 3 thermocycler (Applied Biosystems, Thermo Fisher Scientific). All procedures were performed according to the manufacturer’s recommendations. qPCRs were performed in triplicate. Efficiencies of Cori_fwd/Cori_rev and Ter_fwd/Ter_rev primer pairs were evaluated from a fivefold dilution series of eight different concentrations. Relative abundance of *Cori* to *ter* was calculated using the Pfaffl method, which considers the respective primer set efficiencies (90). The *Cori/ter* ratio of ∆*hmrA* was normalized to that of WT. Three independent biological replicates were used for this quantification.

### Flow cytometry

Quantification of chromosome content at the population level was performed as previously described (89, 91), with few modifications. Overnight cultures in M2X were diluted to OD_600_ = 0.1 and incubated at 30°C until the early exponential phase (0.3–0.4) was reached. Rifampicin was added to the cultures at a final concentration of 15 µg mL^−1^, to block initiation of chromosomal replication and tubes were incubated at 30°C for 3 h (to allow ongoing chromosome replication to be completed). Cells were then fixed by ethanol addition to a final concentration of 70% (vol/vol) and stored at 4°C for 1 h. Cells were stained with 2.5 µM SYTOX Green Nucleic Acid Stain (Invitrogen) and incubated at 4°C overnight. Samples were analyzed using a FACSAria III (BD Life Science) cytometer. Approximately 50,000 cells were analyzed per sample (three samples of three independent biological replicates for both WT and ∆*hmrA* strains).

### Cell cycle-specific protein localization by fluorescence microscopy

To assess cell cycle timing, cells expressing fluorescent fusions to ParB, DivJ, and PleC proteins were tracked by microscopy. The localization and duplication of the chromosome partitioning protein ParB were tracked by time-lapse microscopy using strains harboring a *parB::gfp* fusion (92). To ensure that both WT and ∆*hmrA* strains were subject to the same conditions during the experiment, one strain was labeled with dsRed and the strain that was labeled was switched for each independent replicate. A volume of 1 µL of a mixture of dsRed-labeled/unlabeled cells (OD_600_ = 0.4–0.6) was placed on top of a pad containing 1% SeaKem LE Agarose diluted in M2X and sealed under a coverslip with valap. Time-lapse microscopy images were taken every 2 min for 8 h. Time-lapse movies were visualized in ImageJ (84) to manually assess the timing of ParB duplication (swarmer to stalked cell differentiation), with *t* = 0 defined as the time of division.

The localization of PleC and DivJ was determined in cells harboring both a DivJ-CFP and a PleC-YFP fusion (82), which were synchronized as described above. Synchronized cells were harvested from birth from a synchrony petri dish every 10–15 min and imaged under a 1% SeaKem LE Agarose pad diluted in water. Ten random fields of view were taken for phase contrast, and CFP and YFP fluorescence, for each time point (three independent replicates). Quantification of the number of cells per field of view with a localized PleC-YFP and DivJ-CFP was performed manually.

### Biofilm formation assay in response to the presence of metals or temperature change

To study the metal dose-response of *C. crescentus*, strains were grown overnight in M2X at 30°C with shaking at 180 rpm. Starter cultures were diluted to an OD_600_ of 0.05 in 500 µL of M2X containing various concentrations of metal salts (0–10 µM for CuSO_4_ and 0–100 µM for ZnSO_4_) in 24-well plates. Plates were incubated statically overnight, and biofilm assays were performed as described above.

To study response to temperature, the procedure was the same except that no metal was added to the medium and plates were incubated at various temperatures, as indicated.

### *hfiA* expression assay using β-galactosidase reporter

Strains bearing a transcriptional reporter plasmid for the *hfiA* gene promoter fused to *lacZ* (15) were inoculated into 5 mL of M2X containing 2 µg mL^−1^ tetracycline and incubated at 30°C overnight. Cultures were then diluted in the same culture medium to an OD_600_ of 0.05 and incubated until an OD_600_ of 0.15–0.20 was reached. β-Galactosidase activity was measured colorimetrically as described previously (93). A volume of 200 µL of cells was added to 600 µL of Z buffer (60 mM Na_2_HPO_4_, 40 mM NaH_2_PO_4_, 10 mM KCl, 1 mM MgSO_4_, and 50 mM β-mercaptoethanol), 50 µL of chloroform, and 25 µL of 0.1% (wt/vol) SDS. A volume of 200 µL of substrate *o*-nitrophenyl-β-d-galactoside (ONPG; 4 mg mL^−1^) was then added and time was recorded until the development of a yellow color. The reaction was stopped by adding 400 µL of 1M Na_2_CO_3_. Absorbance at 420 nm (*A*_420_) was measured and the Miller Units of β-galactosidase activity were calculated as (*A*_420_ × 1,000)/((OD_600_ × *t*) × *v*), where *t* is the incubation time in minutes after addition of ONPG, and *v* is the volume of culture (in mL) used in the assay.

### Structure prediction and analysis

Predicted structures of monomeric proteins were retrieved from the AlphaFold Protein Structure Database (29). Structures of protein complexes were predicted using AlphaFold-multimer (32) as implemented through ColabFold (33). Protein structure models were visualized using ChimeraX (94). Predicted structures were submitted to the Dali (95) and Foldseek (30) servers for comparison to experimentally determined structures deposited in the Protein Data Bank.

## SUPPLEMENTAL MATERIAL

Supplemental material mbio.01002-24-s0001.pdf Fig. S1 to S14; Tables S1 and S2.

ASM does not own the copyrights to Supplemental Material that may be linked to, or accessed through, an article. The authors have granted ASM a non-exclusive, world-wide license to publish the Supplemental Material files. Please contact the corresponding author directly for reuse.
