# Supplementary material for: The HmrABCX pathway regulates the transition between motile and sessile lifestyles in Caulobacter crescentus by a mechanism independent of hfiA transcription
Source: mBio. 2024 Sep 4;15(10):e01002-24. doi: 10.1128/mbio.01002-24 (PMC11481889; doi:10.1128/mbio.01002-24)
Supplement: Supplemental material — Fig. S1 to S14; Tables S1 and S2. [file mbio.01002-24-s0001.pdf]

SUPPLEMENTARY DATA

The HmrABCX pathway regulates the transition between motile and sessile lifestyles in *Caulobacter crescentus* by a mechanism independent of *hfiA* transcription

Sébastien Zappa[#], Cécile Berne[#], Robert I. Morton III[#], Gregory B. Whitfield, Jonathan De Stercke, and Yves V. Brun*

Supplementary figures S1 to S14

Supplementary tables S1 and S2

SUPPLEMENTARY FIGURE S1

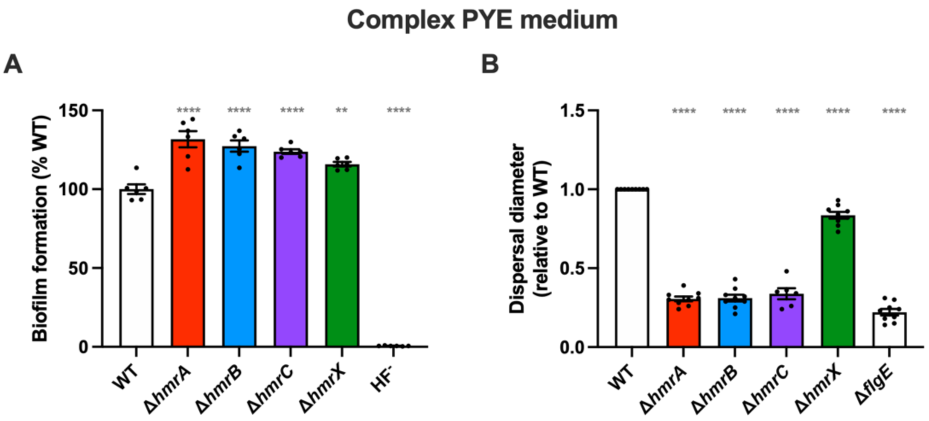

Figure S1: Biofilm and semisolid agar swimming of the *hmr* mutants using complex PYE medium. (A) Biofilm formation in 24-multiwell plates after 24 h of incubation in PYE medium. Results are expressed as a percentage of biofilm formed by each strain compared to WT set at 100%. Results are given as the average of three independent experiments, each run in triplicate, with error shown as SEM. Statistical comparisons are calculated using one sample t-tests to determine if the mean of each sample differs significantly from 100. **(B)** Motility assays through PYE semisolid agar. Swim rings obtained after 5 days of incubation at room temperature were measured and normalized to WT ring diameter measured on the same plate (set to 1). Bar graphs indicate the mean of three independent replicates with SEM run in duplicates. Statistical comparisons to WT are calculated using one sample t-tests to determine if the mean of each sample differs significantly from 1. * $P < 0.05$; **** $P < 0.0001$.

SUPPLEMENTARY FIGURE S2

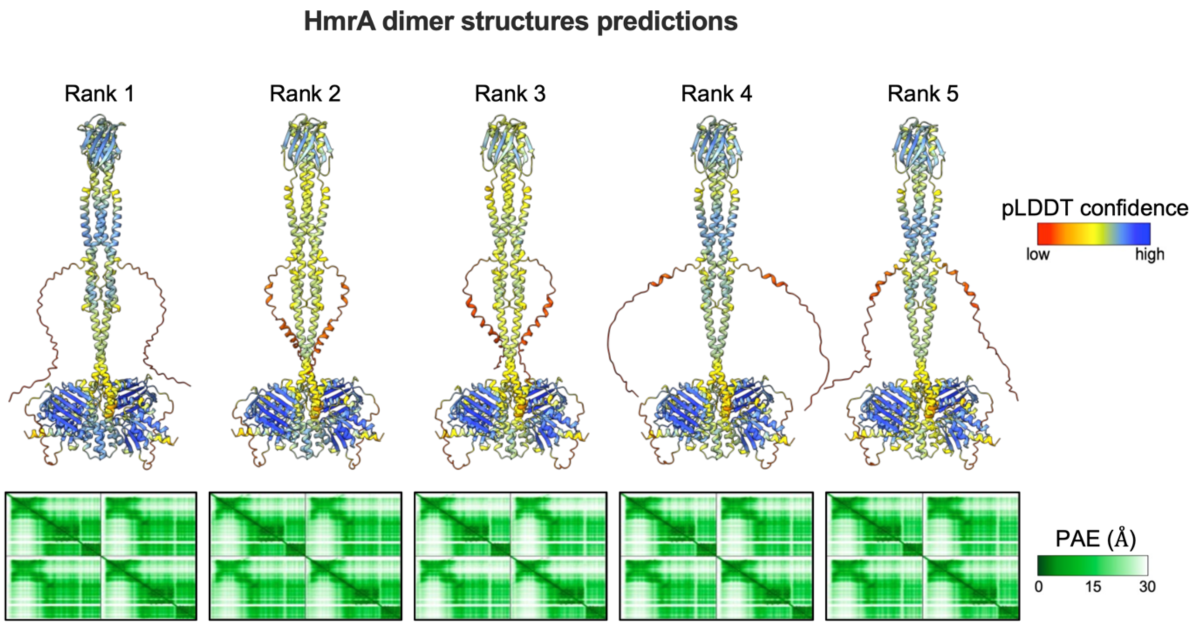

Figure S2: HmrA dimer structures predicted by AlphaFold-multimer as implemented within ColabFold. Structures are aligned and shown from the same orientation. Structures are ranked according to the predicted template modeling (pTM) score and are coloured according to the predicted local distance difference test (pLDDT) score, which indicates per-residue model confidence for the individual protein chains within the complex. Confidence in the prediction of the complex is indicated by the predicted aligned error (PAE) scores, which indicate positional error in angstroms for a given pair of residues across both protein chains.

SUPPLEMENTARY FIGURE S3

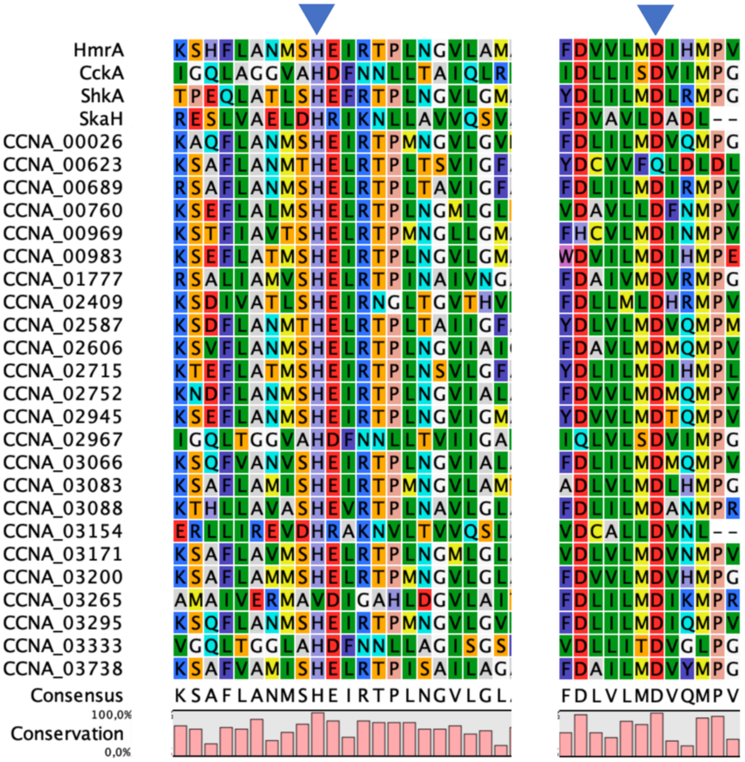

Figure S3: structural analysis of HHKs identified in the *C. crescentus* genome. Protein sequence alignment for the active sites of the 28 predicted HHK present in the *C. crescentus* genome, including the experimentally verified CckA, ShkA and SkaH. Conserved putative catalytical residues, corresponding to H286 and D578 in HmrA, are highlighted with arrows.

SUPPLEMENTARY FIGURE S4

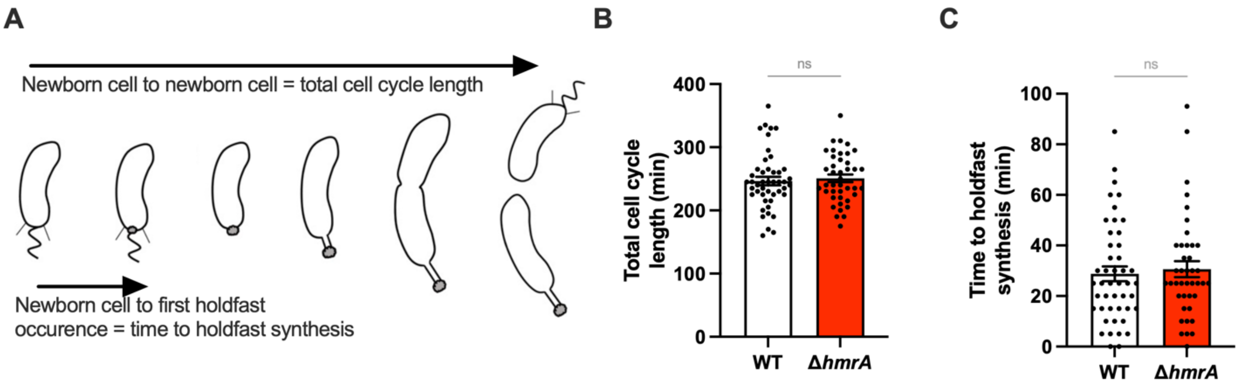

Figure S4: The cell cycle and timing of holdfast synthesis is not affected in a ∆*hmrA* mutant. (A) Representation of total cell cycle length and time to holdfast synthesis, which are measured in panels B and C. **(B)** Timing of total cell cycle length and **(C)** timing of holdfast synthesis by newly divided swarmer cells on M2X agarose pads containing AF488-WGA. The timing between when a new cell divides and when the holdfast first appears on that same cell (B) and when that cell completes an entire cell cycle (A) are recorded. Around 50 cells were counted in three independent replicates. Statistical comparisons are calculated using unpaired *t*-tests (ns = not significant).

SUPPLEMENTARY FIGURE S5

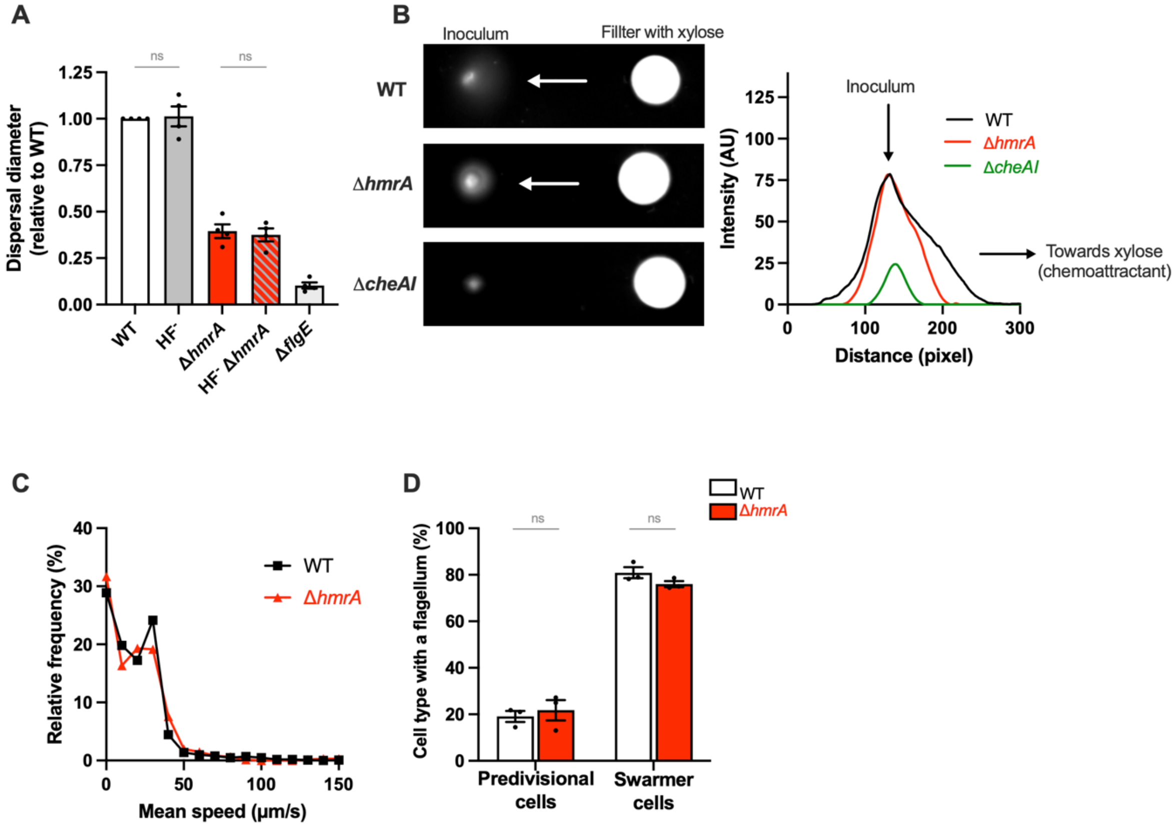

Figure S5: Holdfast is not required for the impaired swimming through semisolid medium of the Δ*hmrA* mutant, and the Δ*hmrA* mutant is not impaired for chemotaxis or for motility in liquid. (A) Motility assays in semisolid agar. Results are normalized to WT (NA1000 *hfsA*+) ring diameter measured on the same plate (set to 1). The HF$^-$ strain (NA1000) does not produce holdfasts. Bar graphs indicate the mean of three independent replicates, and error bars represent SEM. Statistical comparisons are calculated using unpaired *t*-tests (ns: not significant)**. (B)** Chemotaxis assays. Strains are inoculated by spotting into M2 (no carbon) + 0.2% noble agar plate and incubated for 48h. A filter paper containing xylose was placed at a similar distance for the inoculum for each tested strain, to act as a chemoattractant. The relative pixel intensity profile through the center of the chemotaxis ring in a representative image is shown on the right. **(C)** Frequency distribution for swimming run mean speeds. **(D)** Proportion of swarmer and predivisional cells harboring a flagellum. The results are calculated as a percentage of total flagellated cells for each strain (set as 100%) and represent the average of at least three independent replicates (more than 200 flagellated cells per replicate). The error bars represent the SEM. Statistical comparisons are calculated using unpaired *t*-tests (ns: not significant).

SUPPLEMENTARY FIGURE S6

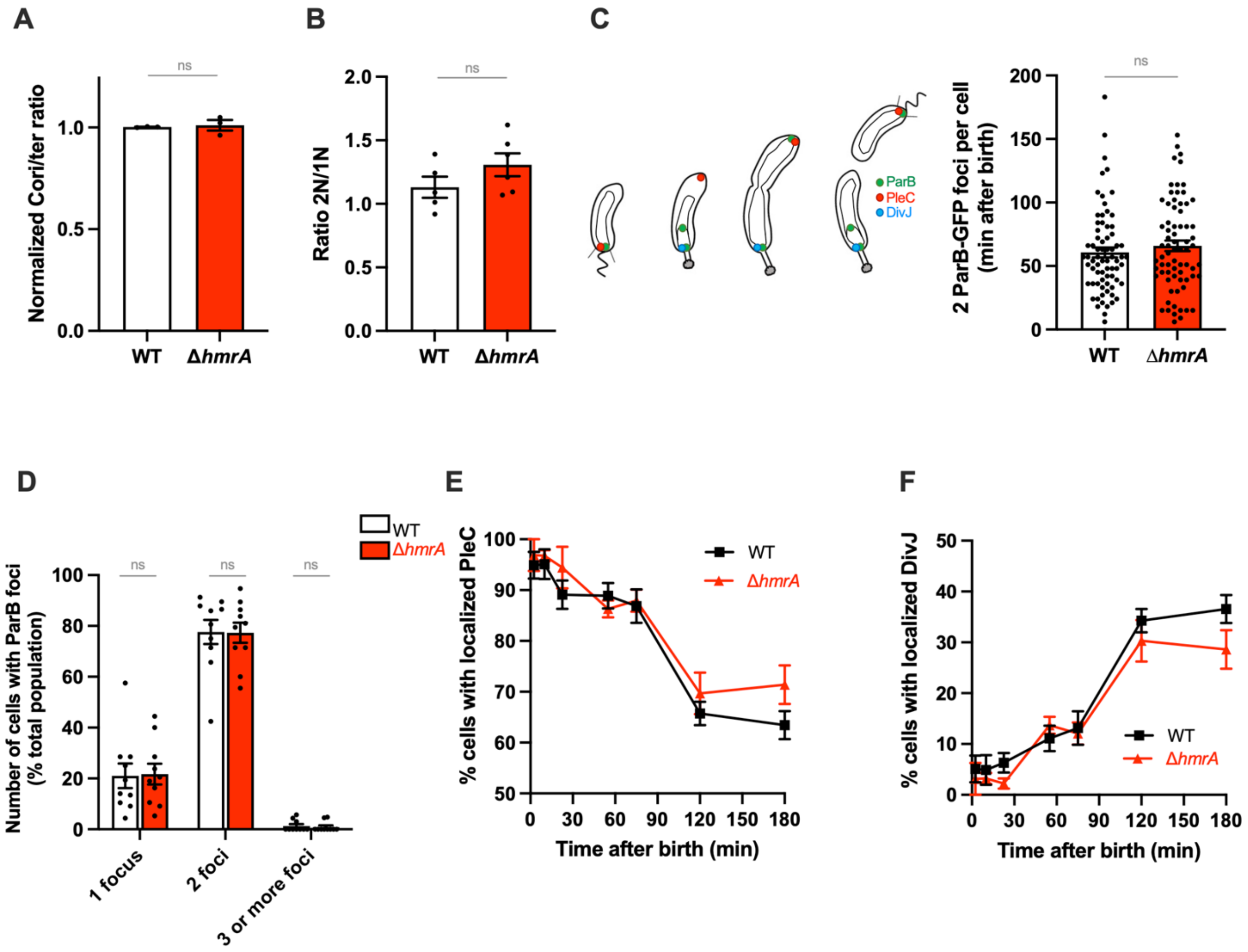

Figure S6: HmrA does not regulate *C. crescentus* chromosome segregation and cell differentiation. (A) Relative *Cori/ter* ratio measured by qPCR. Results are given as the average of three independent replicates, normalized for WT in each replicate, and the error represents the SEM. **(B)** Quantification of chromosome content of rifampicin-treated cells harboring. The ratio of 2 chromosomes (2N) over one chromosome (1N) was measured by flow cytometry. The bars represent the average of three samples and three independent replicates, with 50,000 cells for each sample and the error bars representing the SEM. **(C)** Timing of duplication of ParB. Schematic representation of ParB, PleC and DivJ localization during the cell cycle (left). Cells harboring a ParB-GFP fusion were tracked over time, and the first appearance of a second ParB protein was recorded, from the time of birth of each cell (right). Approximately 100 cells were analyzed in three independent replicates. **(D)** Number of cells with a 1 or 2 ParB foci at the population level. Cells grown to mid-exponential phase (OD600 = 0.4-0.6) were imaged by fluorescence microscopy, and the number of cells with 1, 2 or more ParB foci par field of view was calculated. Only cells exhibiting a fluorescent ParB signal are reported. Quantification for WT and Δ*hrmA* are shown in white and red respectively. **(E)** Number of cells with a localized PleC protein (indicative of a swarmer cell) over time. Synchronized cells were imaged by fluorescence microscopy and the number of cells per field of view with a localized PleC-YFP focus were quantified. The points are the average of 10 random images (around 100 cells) and three independent replicates for each time point, and the error bars represent the SEM. Data for WT and Δ*hmrA* are shown in black squares and red triangles respectively. **(F)** Number of cells with a localized DivJ protein (indicative of a stalked cell) over time. Synchronized cells were imaged by fluorescence microscopy and the number of cells per field of view with a localized DivJ-CFP focus was quantified. The points are the average of 10 random images (around 100 cells) and three independent replicates for each time point, and the error bars represent the SEM. Data for WT and Δ*hrmA* are shown in black squares and red triangles respectively. Statistical comparisons are calculated using unpaired *t*-tests (ns: not significant)**.**

SUPPLEMENTARY FIGURE S7

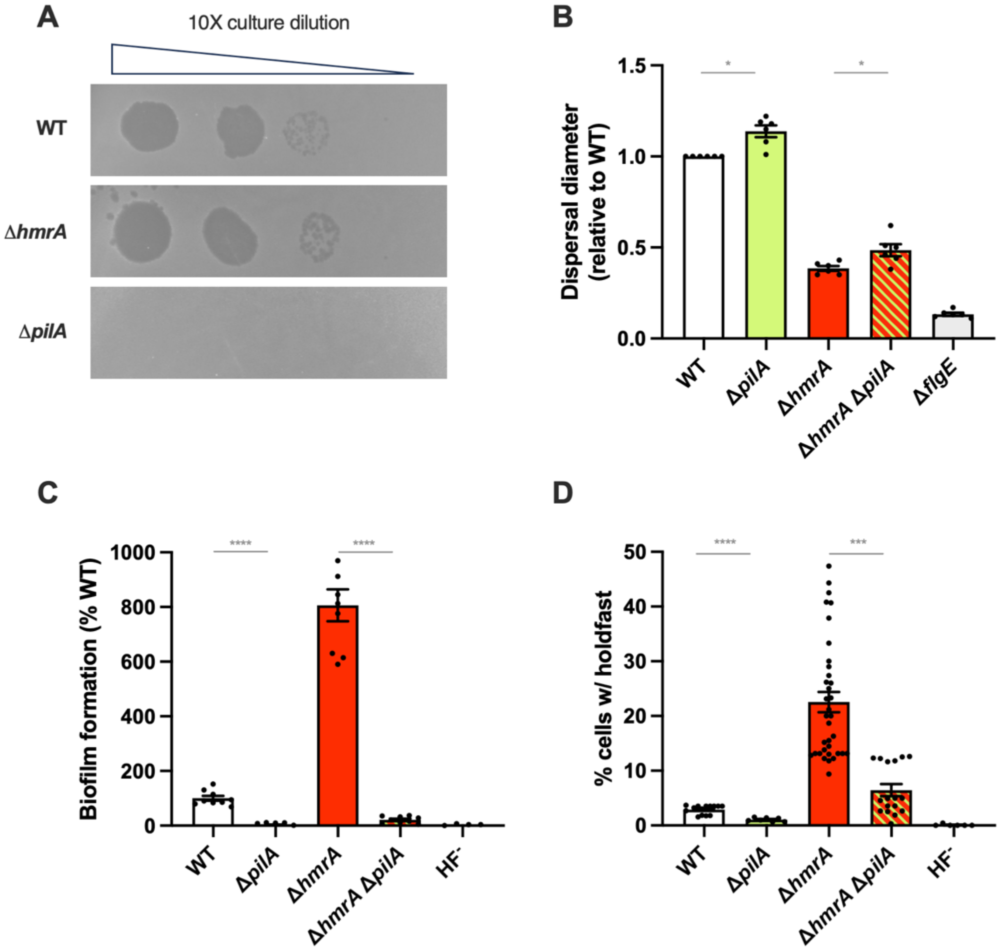

Figure S7: Pili are not linked to the HmrA regulation pathway. (A) ΦCbK phage sensitivity assays. Phage were diluted to 10^{-8} pfu per ml. Ten µl of the dilutions were spotted onto a PYE top agar plate containing cells of each strain. The plate was imaged after 24-hour incubation. **(B)** Motility assays through M2X semisolid agar. Swim rings obtained after 5 days of incubation at room temperature were measured and normalized to WT ring diameter measured on the same plate (set to 1). Bar graphs indicate the mean of three independent replicates, and error bars represent SEM. **(C)** Biofilm formation in 24-multiwell plates after 24 h of incubation in M2X medium. Results are expressed as a percentage of biofilm formed by each strain compared to WT set at 100%. Results are given as the average of three independent experiments, each run in triplicate, with error bars representing the SEM. **(D)** Quantification of cells harboring a holdfast in mixed populations. The results represent the average of at least three independent replicates (more than 300 cells per replicate) and the error bars represent the SEM. For all the graphs, statistical comparisons between WT and Δ*pilA,* and between Δ*hmrA* and Δ*hmrA* Δ*pilA,* respectively, are calculated using unpaired *t*-tests (* $P < 0.05$; *** $P < 0.001$; **** $P < 0.0001$).

SUPPLEMENTARY FIGURE S8

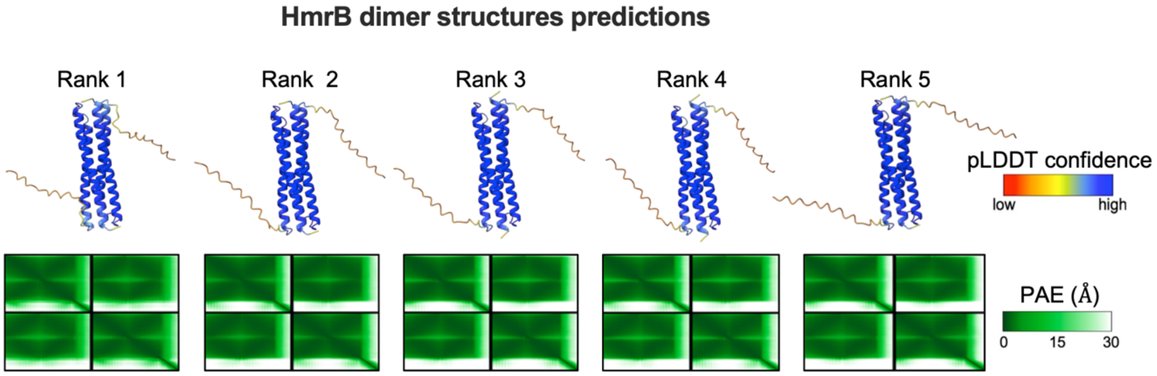

Figure S8: HmrB dimer structures predicted by AlphaFold-multimer as implemented within ColabFold. Structures are aligned and shown from the same orientation. Structures are ranked according to the predicted template modeling (pTM) score and are colored according to the predicted local distance difference test (pLDDT) score, which indicates per-residue model confidence for the individual protein chains within the complex. Confidence in the prediction of the complex is indicated by the predicted aligned error (PAE) scores, which indicate positional error in angstroms for a given pair of residues across both protein chains.

SUPPLEMENTARY FIGURE S9

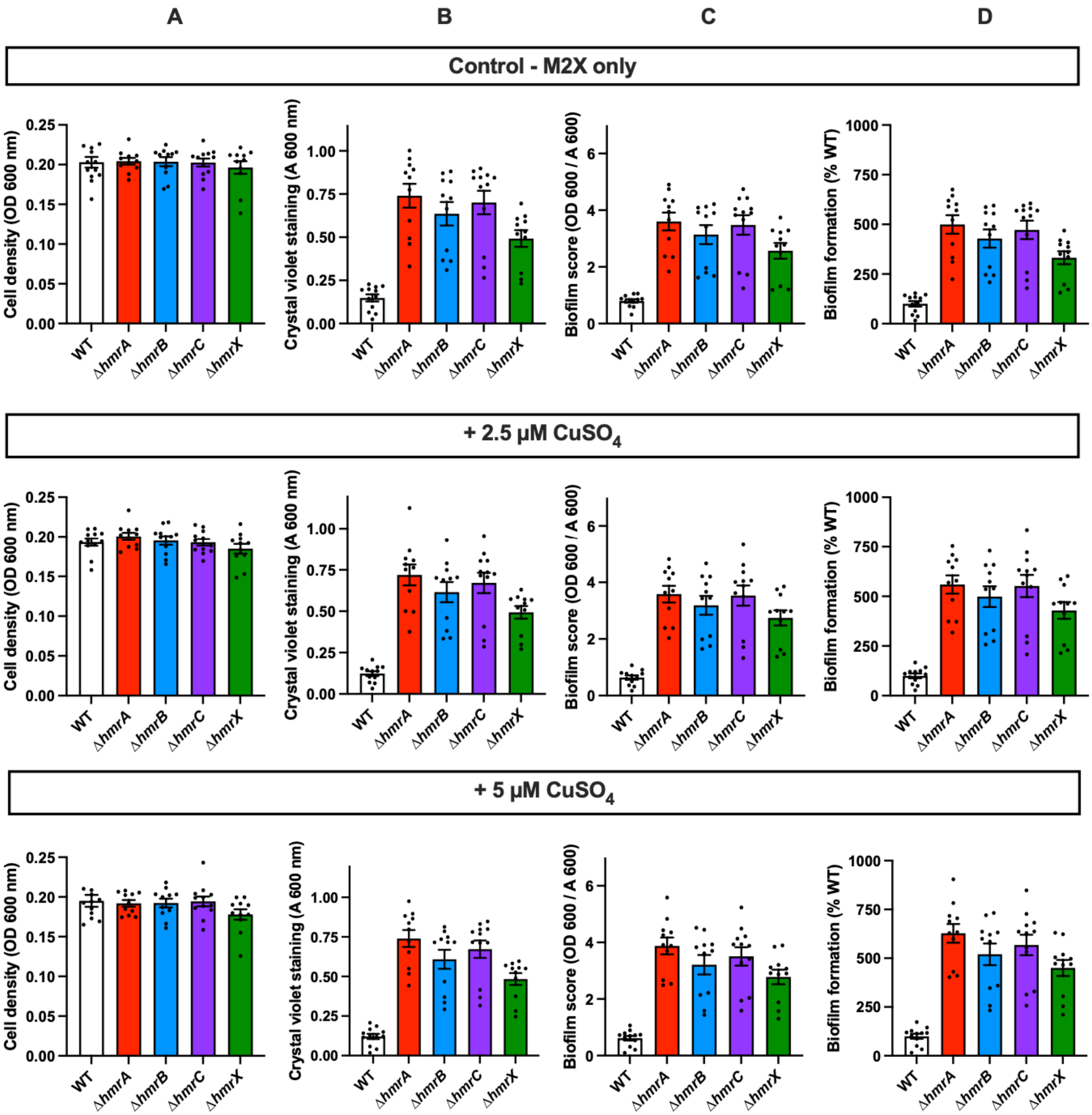

SUPPLEMENTARY FIGURE S9 (continued)

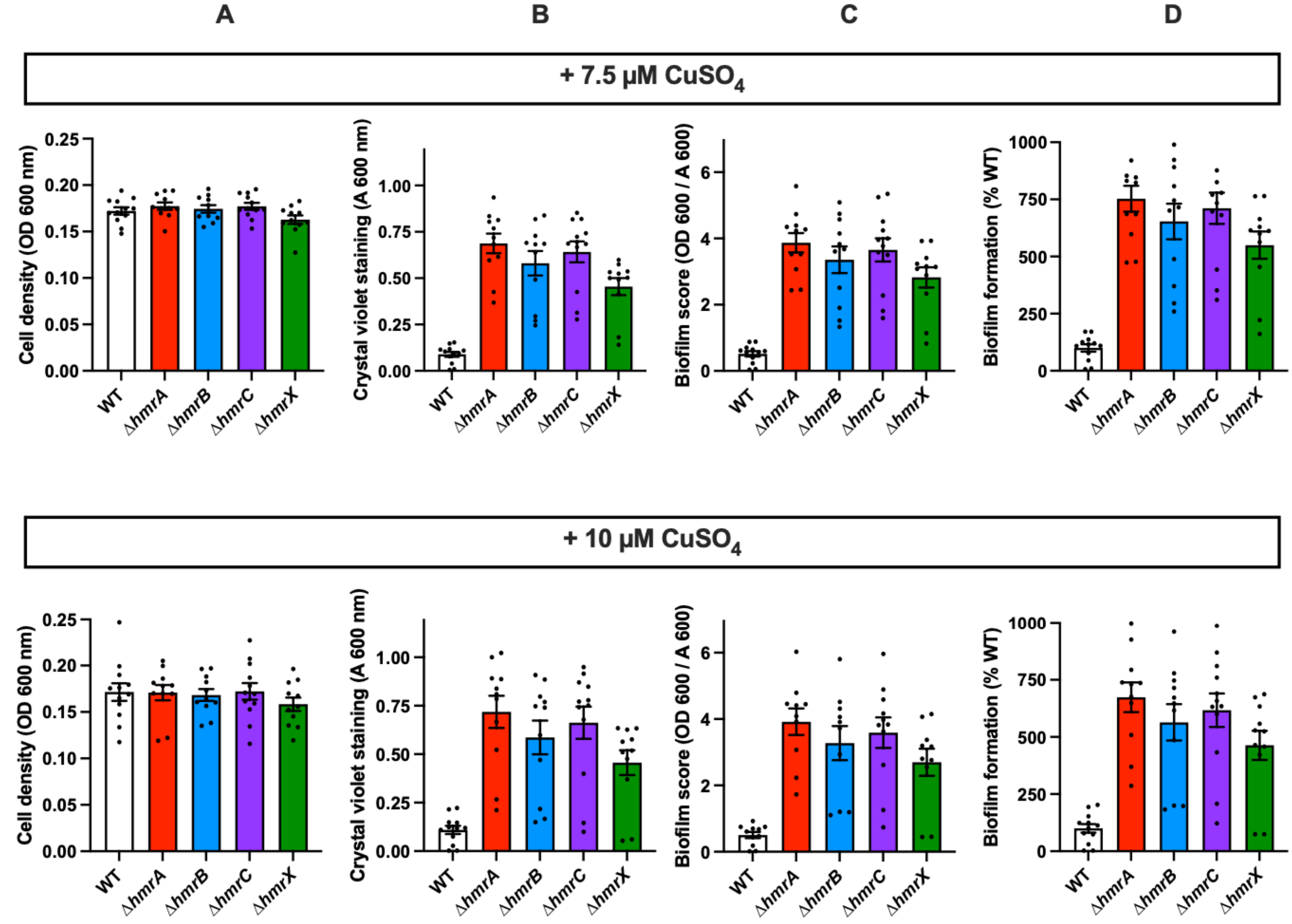

Figure S9: growth and biofilm formation in the presence of different concentrations of $CuSO_4$. S amples were grown in 24-multiwell plates in M2X medium with different $CuSO_4$ concentrations (0, 2.5, 5, 7.5, and 10 µM). For each metal concentration, the total growth in each well **(A)** and total attached biomass stained using crystal violet **(B)** were measured after 24 h of incubation and used to calculate the biofilm score (attached biomass / total growth) **(C)** and percentage of biofilm formed compared to WT set at 100% **(D)** Results are given as the average of at least four independent replicates run in duplicates, with SEM.

SUPPLEMENTARY FIGURE S10

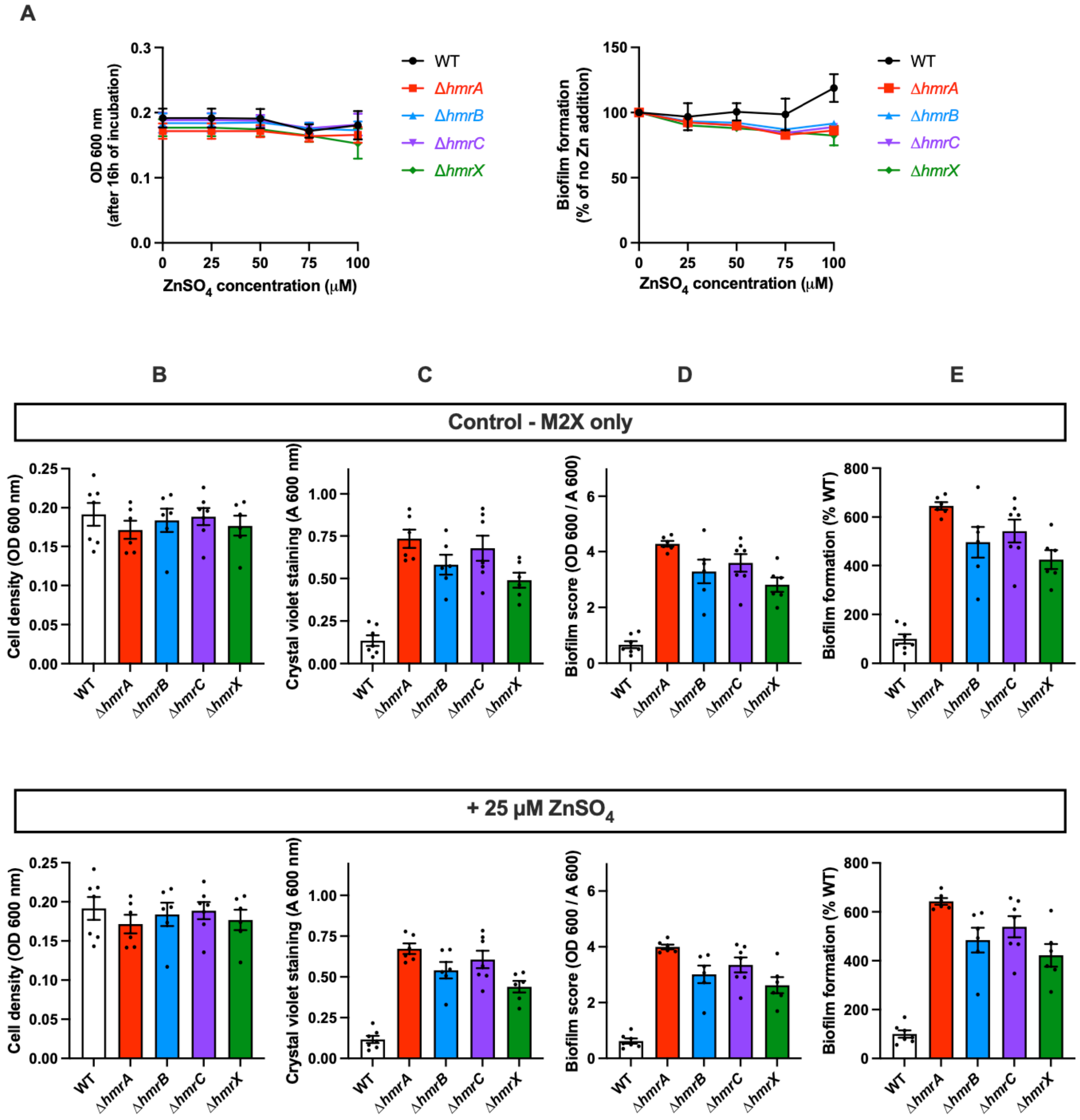

SUPPLEMENTARY FIGURE S10 (continued)

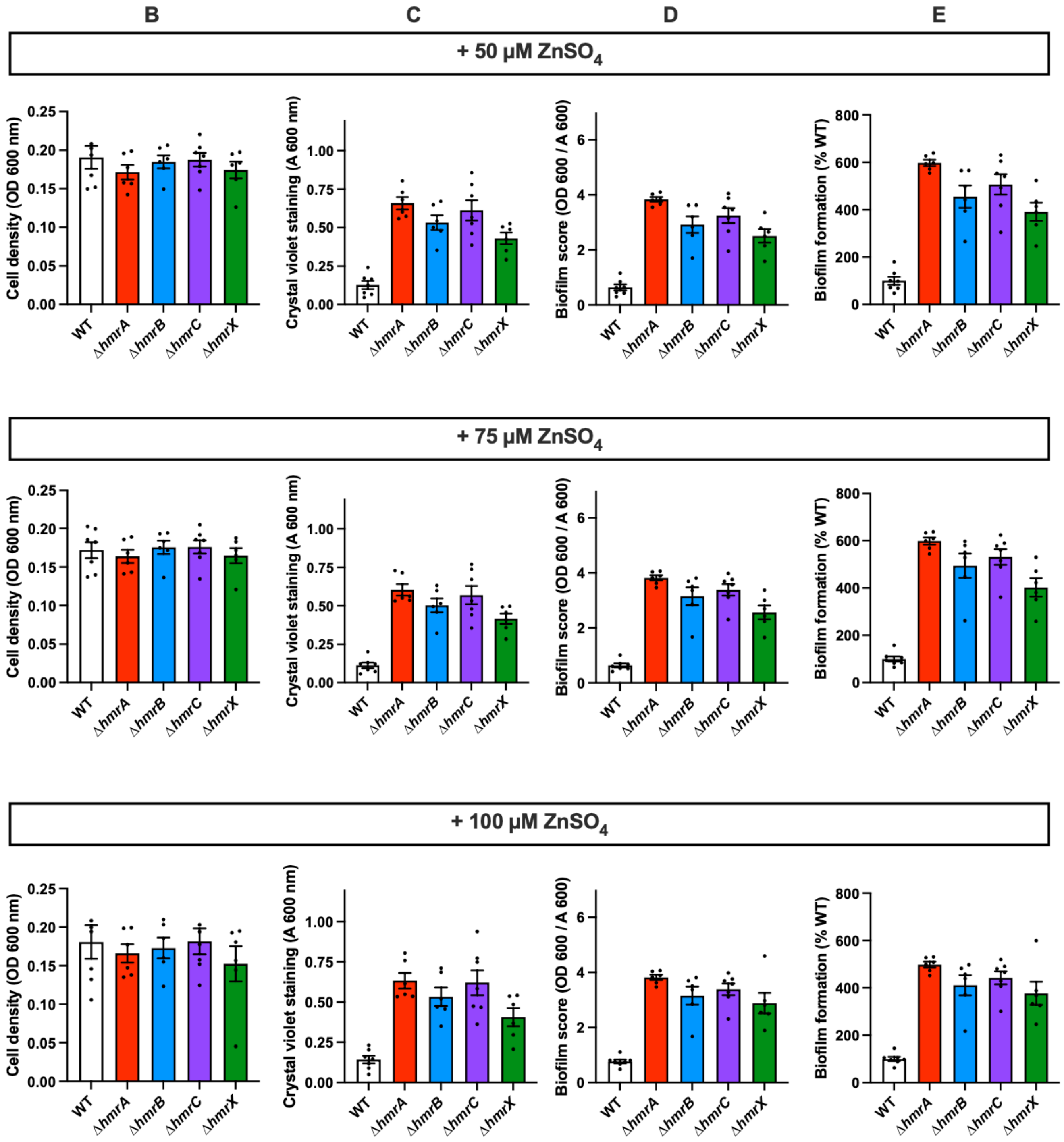

Figure S10: Growth and biofilm formation in the presence of different concentrations of $ZnSO_4$. Samples were grown in 24-multiwell plates in M2X medium with different $ZnSO_4$ concentrations (0, 25, 50, 75, and 100 µM). **(A)** Overnight growth (left) and biofilm formation (right) for WT (black circles), Δ*hmrA* (red squares), Δ*hmrB* (blue triangles), Δ*hmrC* (purple down triangles), and Δ*hmrX* (green diamonds). Samples were grown in 24-multiwell plates for after 24 h of incubation in M2X medium, with different concentration of $ZnSO_4$. Biofilm results

are expressed as a percentage of biofilm formed by each strain in the presence of metal compared to the no metal addition set at 100%. Results are given as the average of eight independent experiments, each run in triplicate, and the error bars represent SEM. Unprocessed data are shown in panels B to F: for each metal concentration, the total growth in each well **(B)** and total attached biomass stained using crystal violet **(C)** were measured after 24 h of incubation and used to calculate the biofilm score (attached biomass / total growth) **(D)** and percentage of biofilm formed compared to WT set at 100% **(E)**. Results are given as the average of at least three independent replicates run in duplicates, with SEM.

SUPPLEMENTARY FIGURE S11

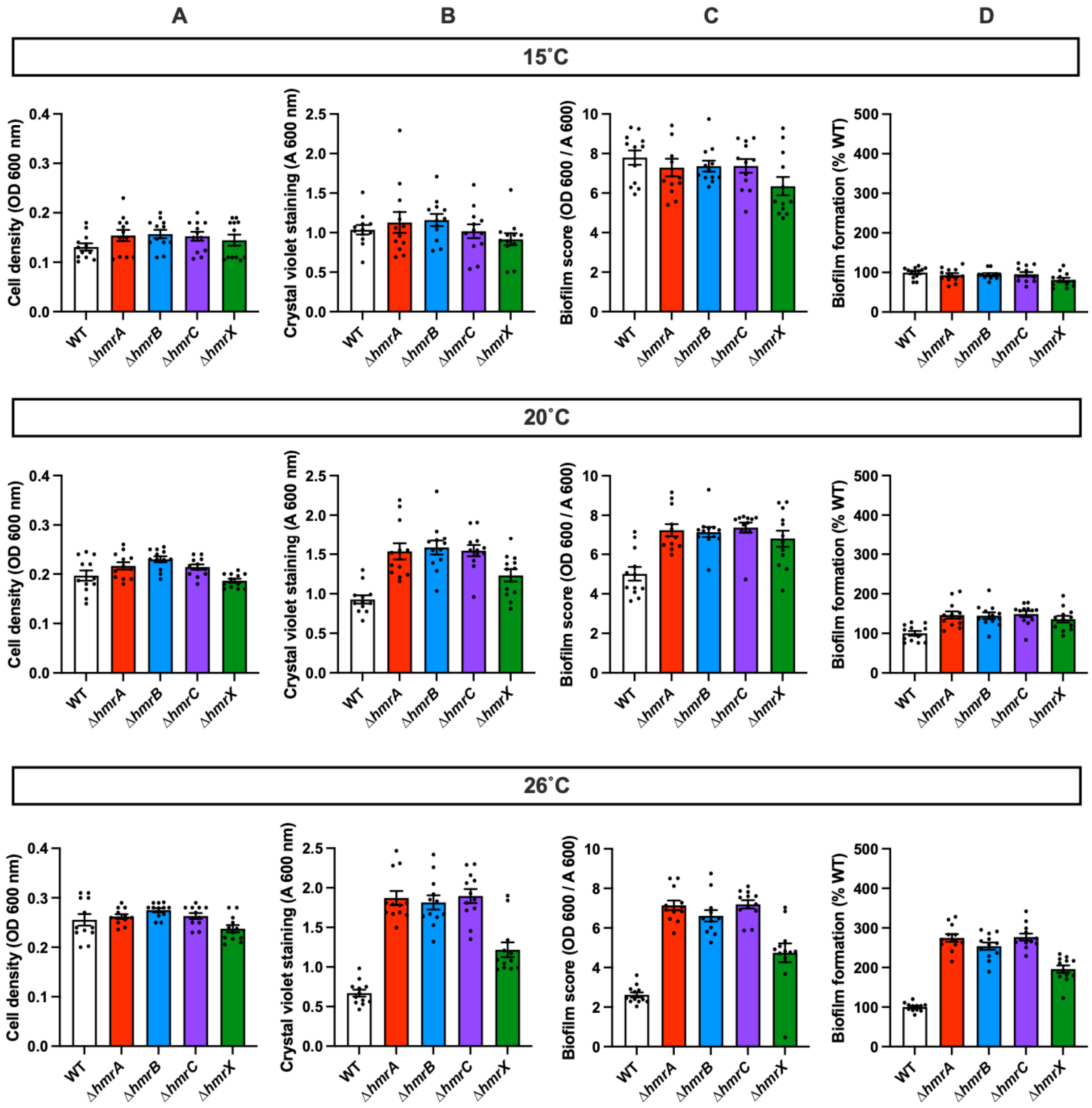

SUPPLEMENTARY FIGURE S11 (continued)

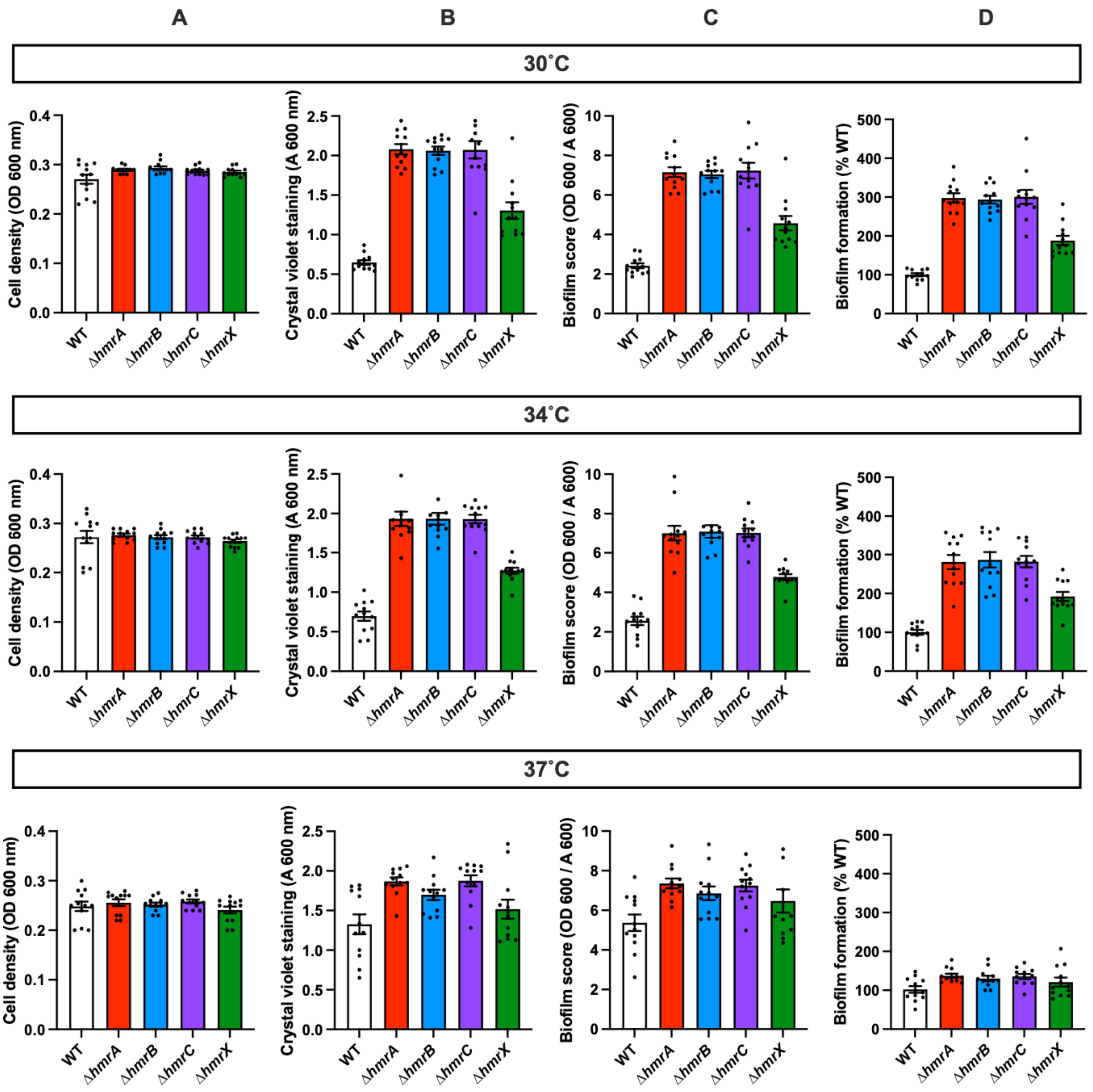

Figure S11: Growth and biofilm formation at different temperatures. Samples were grown in 24-multiwell plates in M2X medium at different temperatures (15˚C, 20˚C, 26˚C, 30˚C, 34˚C, and 37˚C). For each temperature, the total growth in each well **(A)** and total attached biomass stained using crystal violet **(B)** were measured after 24 h of incubation and used to calculate the biofilm score (attached biomass / total growth) **(C)** and percentage of biofilm formed compared to WT set at 100% **(D)**. Results are given as the average of six independent replicates run in duplicates, and the error bars represent the SEM.

SUPPLEMENTARY FIGURE S12

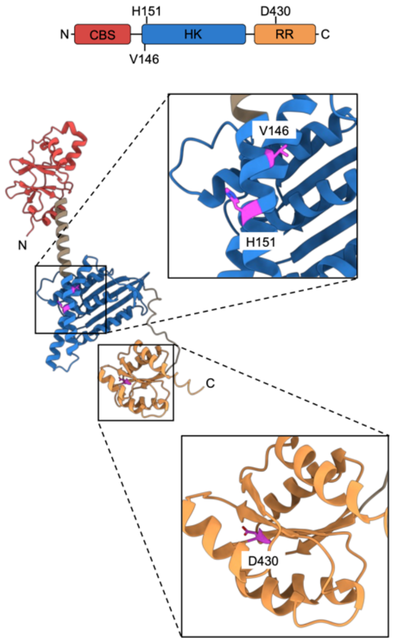

Figure S12:_Prediction of three-dimensional structure of *CCNA_03265* encoded HHK using AlphaFold. V146 is the residue that aligns with the putative catalytic histidine of other HHKs (Fig. S3). H151 is a candidate as catalytic histidine residue. Putative catalytic aspartate, D430, is also displayed.

SUPPLEMENTARY FIGURE S13

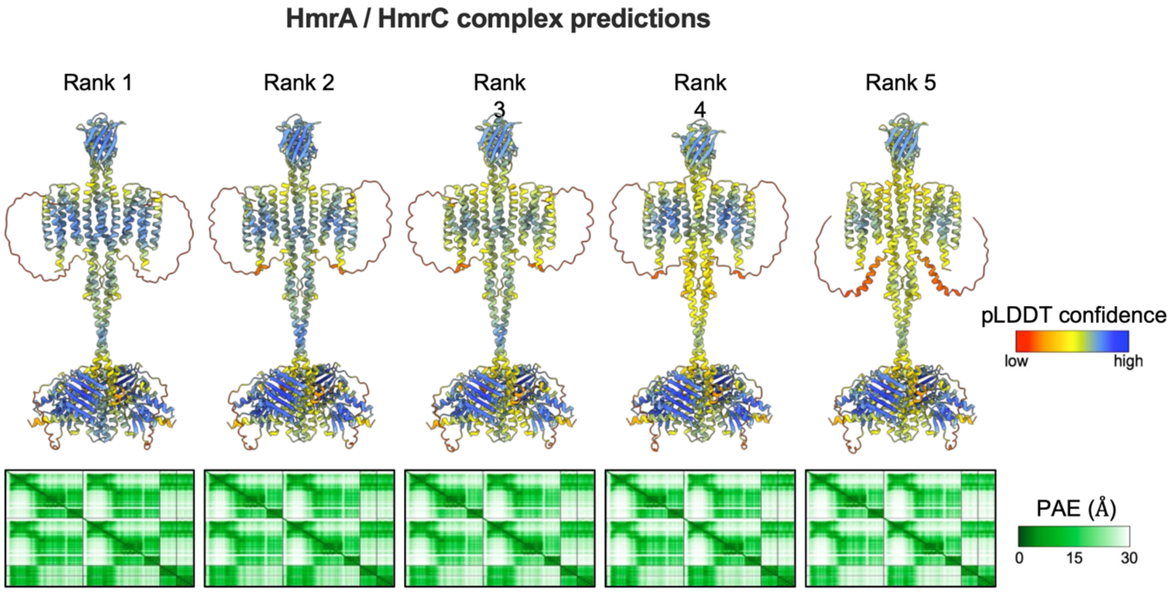

Figure S13: Structures of a HmrA dimer in complex with two copies of HmrC, as predicted by AlphaFold-multimer implemented within ColabFold. Structures are aligned and shown from the same orientation. Structures are ranked according to the predicted template modeling (pTM) score and are colored according to the predicted local distance difference test (pLDDT) score, which indicates per-residue model confidence for the individual protein chains within the complex. Confidence in the prediction of the complex is indicated by the predicted aligned error (PAE) scores, which indicate positional error in angstroms for a given pair of residues across both protein chains.

SUPPLEMENTARY FIGURE S14

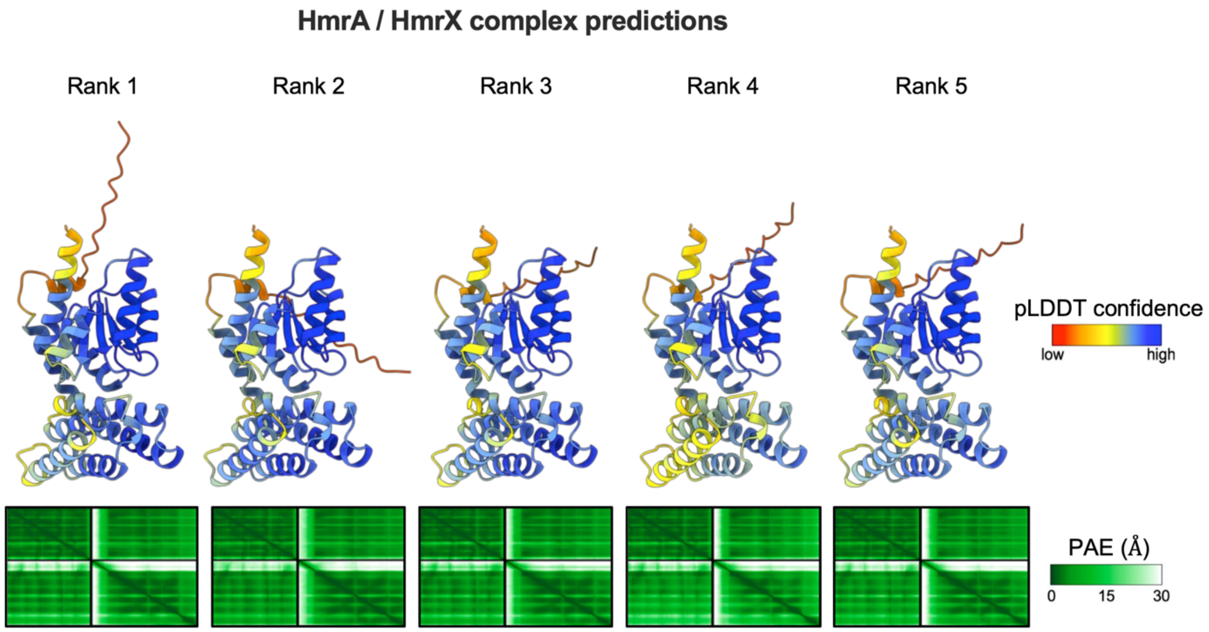

Figure S14: Structures of the response regulator domain of HmrA in complex with HmrX, as predicted by AlphaFold-multimer implemented within ColabFold. Structures are aligned and shown from the same orientation. Structures are ranked according to the predicted template modeling (pTM) score and are colored according to the predicted local distance difference test (pLDDT) score, which indicates per-residue model confidence for the individual protein chains within the complex. Confidence in the prediction of the complex is indicated by the predicted aligned error (PAE) scores, which indicate positional error in angstroms for a given pair of residues across both protein chains.

SUPPLEMENTARY TABLE S1: *Caulobacter crescentus* strains used in this study

Strain	Description or construction	Source or reference
YB5801 (FC764)	NA1000 *hfsA*+ WT	(96)
YB127	NA1000	(97)
YB6927	NA1000 *hfsA*+ Δ*hmrA (CCNA_03326)*	This study
YB6928	NA1000 *hfsA*+ Δ*hmrB (CCNA_03325)*	This study
YB6934	NA1000 *hfsA*+ Δ*hmrC (CCNA_03324)*	This study
YB6935	NA1000 *hfsA*+ Δ*03327*	This study
YB6569	NA1000 *hfsA*+ WT pMR10	This study
YB6570	NA1000 *hfsA*+ Δ*hmrA* pMR10	This study
YB6966	NA1000 *hfsA*+ Δ*hmrA* pMR10-*hmrA*	This study
YB5679	NA1000 *hfsA*+ Δ*hmrA* pMR10-*hmrA* H286A	This study
YB7916	NA1000 *hfsA*+ Δ*hmrA* pMR10-*hmrA* D578A	This study
YB8897	NA1000 *hfsA*+ Δ*hmrA Tn7::hmrA*	This study
YB6375	NA1000 *hfsA*+ Δ*flgE*	(37)
YB4643	CB15 Δ*cheAI*	(16)
YB6562	NA1000 *hfsA*$^+$ WT FljKT176C	(37)
YB6578	NA1000 *hfsA*$^+$ Δ*hmrA* FljKT176C	This study
YB4748	CB15 WT *miniTn7::dsred*	(81)
YB6010	NA1000 *hfsA*$^+$ WT ::*miniTn7dsred* FljKT176C	This study
YB6013	NA1000 *hfsA*$^+$ Δ*hmrA* ::*miniTn7dsred* FljKT176C	This study
YB7370	NA1000 *hfsA*+ Δ*dgcB*	This study
YB8826	NA1000 *hfsA*+ Δ*pleD*	This study
YB8898	NA1000 *hfsA*+ Δ*hmrA* Δ*dgcB*	This study
YB8899	NA1000 *hfsA*+ Δ*hmrA* Δ*pleD*	(37)
YB8011	NA1000 *hfsA*+ Δ*hmrX*	This study
YB8900	NA1000 *hfsA*+ Δ*hmrX Tn7::hmrX*	This study
YB8901	NA1000 *hfsA*+ Δ*hmrA* Δ*hmrX*	This study
YB8902	NA1000 *hfsA*+ Δ*hmrA* Δ*hmrB*	This study
YB8903	NA1000 *hfsA*+ Δ*hmrB Tn7::hmrB*	This study
YB6575	NA1000 *hfsA*+ Δ*hmrB* pMR10	This study
YB6576	NA1000 *hfsA*+ Δ*hmrB* pMR10-*hmrB*	This study
YB6008	NA1000 *hfsA*+ Δ*hmrB* pMR10-*hmrB* H50A	This study
YB7918	NA1000 *hfsA*+ Δ*hmrB* pMR10-*hmrB* H74A	This study
YB8904	NA1000 *hfsA*+ Δ*hmrC Tn7::hmrC*	This study
YB10176	NA1000 *hfsA*+ Δ*hmrA* Δ*hmrC*	This study
YB7844	NA1000 *hfsA*+ Δ*hfiA*	This study
YB10177	NA1000 *hfsA*+ Δ*hmrA* Δ*hfiA*	This study
YB7843	NA1000 *hfsA*+ Δ*hmrA* pRKlac290	This study
YB7836	NA1000 *hfsA*+ WT pRKlac290-P_{hfiA}	This study
YB8893	NA1000 *hfsA*+ Δ*hmrA* pRKlac290-P_{hfiA}	This study
YB8894	NA1000 *hfsA*+ Δ*hmrB* pRKlac290-P_{hfiA}	This study
YB8895	NA1000 *hfsA*+ Δ*hmrC* pRKlac290-P_{hfiA}	This study
YB8896	NA1000 *hfsA*+ Δ*hmrX* pRKlac290-P_{hfiA}	This study
YB8819	CB15 Δ*flgE* pRKlac290-P_{hfiA}	(37)
YB9269	CB15 Δ*cheAI* pRKlac290-P_{hfiA}	(16)
YB6374	NA1000 *hfsA*+ Δ*pilA*	(36)
YB10178	NA1000 *hfsA*+ Δ*hmrA* Δ*pilA*	This study
YB5662	NA1000 *hfsA*+ WT *parB-gfp*	This study
YB5664	NA1000 *hfsA*+ Δ*hmrA parB-gfp*	This study
YB5665	NA1000 *hfsA*+ WT *miniTn7::dsred parB-gfp*	This study
YB5667	NA1000 *hfsA*+ Δ*hmrA miniTn7::dsred parB-gfp*	This study
YB385	CB15N *divJ-cfp*, *pleC-yfp*	(82)
YB7099	NA1000 *hfsA*+ WT *divJ-cfp*, *pleC-yfp*	This study
YB7101	NA1000 *hfsA*+ Δ*hmrA divJ-cfp*, *pleC-yfp*	This study

SUPPLEMENTARY TABLE S2: Plasmids used in this study

Plasmid	Description	Antibiotic	Reference
pNPTS138	Litmus 38 derivative used for unmarked deletions in *C. crescentus (sac*B counter-selection)	Kan	(41)
pNPTS139	Litmus 39 derivative used for unmarked deletions in *C. crescentus (sac*B counter-selection)	Kan	(41)
pNPTS*hmrA*	pNPTS138 containing around 500 bp fragments upstream and downstream of *hmrA* (for in-frame deletion)	Kan	This study
pNPTS*hmrB*	pNPTS138 containing around 500 bp fragments upstream and downstream of *hmrB* (for in-frame deletion)	Kan	This study
pNPTS*hmrC*	pNPTS138 containing around 500 bp fragments upstream and downstream of *hmrC* (for in-frame deletion)	Kan	This study
pNPTS*hmrX*	pNPTS138 containing around 500 bp fragments upstream and downstream of *hmrX* (for in-frame deletion)	Kan	This study
pNPTS*dgcB*	pNPTS138 containing around 500 bp fragments upstream and downstream of *dgcB* (for in-frame deletion)	Kan	This study
pNPTS*hfiA*	pNPTS138 derivative used for in-frame deletion of *hfiA*		(15)
pNPTS*fljK*T176C	pNPTS139 containing 753-bp of *fljK* with a T to C point mutation at 103 bp from the start codon	Kan	(37)
pRKlac290	plasmid for *lacZ* transcriptional fusions	Tc	(98)
pAF427	pRKlac290-P*hfiA*	Tc	(15)
pMR10	Mid copy replicating plasmid, IPTG inducible (constitutive in *C. crescentus*)	Kan	(99)
pMR10-*hmrA*	constitutive expression of *hmrA*	Kan	This study
pMR10-*hmrA* H286A	constitutive expression of *hmrA*H286A	Kan	This study
pMR10-*hmrA* D578A	constitutive expression of *hmrA*D578A	Kan	This study
pMR10-*hmrB*	constitutive expression of *hmrB*	Kan	This study
pMR10-*hmrB* H50A	constitutive expression of *hmrB*H50A	Kan	This study
pMR10-*hmrB* H74A	constitutive expression of *hmrB*H74A	Kan	This study
pMR10-*hmrC*	constitutive expression of *hmrC*	Kan	This study
pUC18-mini-Tn7T-*lac*	mini-Tn7 vector for insertion at theTn7 *att* site	Gm	(78)
pTNS3	helper plasmid for mating pTn7 in *C. crescentus*	Ap	(79)
pUC18-mini-Tn7T-*hmrA*	for *hmrA* insertion (native promoter) at theTn7 *att* site	Gm	This study
pUC18-mini-Tn7T-*hmrB*	for *hmrB* insertion (native promoter) at theTn7 *att* site	Gm	This study
pUC18-mini-Tn7T-*hmrC*	for *hmrC* insertion (native promoter) at theTn7 *att* site	Gm	This study
pUC18-mini-Tn7T-*hmrX*	for *hmrX* insertion (native promoter) at theTn7 *att* site	Gm	This study
pFD1	*Mariner*-based mini-transposon vector for random transposon-mediated mutagenesis	Kan	(22)
